# Exosome-derived circ_0001785 delays atherogenesis through the ceRNA network mechanism of miR-513a-5p/TGFBR3

**DOI:** 10.1186/s12951-023-02076-x

**Published:** 2023-10-04

**Authors:** Xiao Tong, Xuan Dang, Dongmei Liu, Ning Wang, Miao Li, Jianbin Han, Jinjin Zhao, Yueqing Wang, Meijiao Huang, Yanliang Yang, Yuhang Yang, Weili Wang, Yan Kou, Junjie Kou

**Affiliations:** 1 Department of Cardiology, The 2nd Affiliated Hospital of Harbin Medical University, 148 Health Care Road, Harbin, Heilongjiang China; 2 The Key Laboratory of Myocardial Ischemia, Chinese Ministry of Education, Harbin, 150000 Heilongjiang China; 3 Department of Ultrasound, The 2nd Affiliated Hospital of Harbin Medical University, Harbin, Heilongjiang China

**Keywords:** Diagnostic marker, Plasma exosome, circRNA, Atherogenesis, ceRNA

## Abstract

**Purpose:**

Endothelial cell dysfunction is a major cause of early atherosclerosis. Although the role of extracellular vesicles in stabilizing atherosclerotic plaques is well established, the effect of circulating exosomes on plaque formation is still unknown. Here, we explored the effect of exosomes on atherosclerosis based on the function that exosomes can act on intercellular communication.

**Patients and methods:**

We extracted serum exosomes from the blood of CHD patients (CHD-Exo) and healthy individuals (Con-Exo). The obtained exosomes were co-cultured with human umbilical vein endothelial cells (HUVECs) in vitro. In addition, we determined that circ_0001785 functions as a competing endogenous RNA (ceRNAs) in coronary artery disease by dual luciferase reporter gene analysis. The protective effect of circ_0001785 against endothelial cell injury was also verified using over-expression lentiviral transfection functional assays. In vivo experiments, we injected over-expressed circ_0001785 lentivirus into the tail vein of mice to observe its therapeutic effect on a mouse model of atherosclerosis.

**Results:**

The vitro co-cultured results showed that the amount of plasma-derived exosomes have an increase in patients with coronary artery disease, and the inflammation and apoptosis of endothelial cells were exacerbated. Over-expression of circ_0001785 reduced endothelial cell injury through the ceRNA network pathway of miR-513a-5p/TGFBR3. Quantitative reverse transcription-polymerase chain reaction identified that the expressed amount of circ_0001785 was reduced in the circulating peripheral blood of CHD patients and increased within human and mouse atherosclerotic plaque tissue. The results of in vivo experiments showed that circ_0001785 reduced aortic endothelial cell injury and the formation of intraplaque neo-vascularization, and enhanced left ventricular diastolic function, thereby delaying the development of atherosclerosis in mice.

**Conclusion:**

Our results demonstrated a new biomarker, exosome-derived circ_0001785, for atherogenesis, which can reduce endothelial cell injury and thus delay atherogenesis through the miR-513a-5p/TGFBR3 ceRNA network mechanism, providing an exosome-based intervention strategy for atherosclerosis.

**Supplementary Information:**

The online version contains supplementary material available at 10.1186/s12951-023-02076-x.

## Introduction

Acute coronary syndrome (ACS) is a series of cardiovascular emergencies caused by coronary flow obstruction and acute myocardial ischemia (AMI). Coronary flow obstruction is mainly due to plaque rupture or erosion caused by atherosclerosis [1–3]. However, a key factor in plaque erosion and rupture is vascular inflammation caused by endothelial cell dysfunction [4]. Due to the specific function of endothelial cells as a barrier between blood vessels and tissues, it can maintain intravascular homeostasis by regulating vascular tone, as well as antithrombotic and anti-inflammatory effects [5]. However, the mechanisms leading to mechanisms of endothelial cell dysfunction are complex and therefore still need to be explored.

Cellular communication is essential to the physiological and pathological processes of almost all diseases, including atherosclerosis. Exosomes are a class of small vesicular structures that can participate in intercellular communication. It can perform biological functions through the different biomolecules it contains, such as DNA, RNA, proteins, lipids or metabolites. Thus, during atherogenesis, it can facilitate communication and adhesion between blood and resident cells of the vessel wall, involving endothelial dysfunction, apoptosis, inflammatory signaling and plaque rupture [6–11].

Here, we studied the mechanism by that circRNAs affect atherogenesis through delivery. Circular RNAs (circRNAs) are a group of noncoding RNAs with a covalent closed-loop structure. resistance to degradation by nucleic acid exonucleases allows their massive and stable expression, which is important for clinical studies [12–17]. It has been well demonstrated that circRNAs have important effects on endothelial cell proliferation, migration, and neovascularization in atherosclerosis [18–20]. In addition, exosome-derived circRNAs have also shown potential applications as disease biomarkers and novel therapeutic targets [21]. Based on exosomes as gene delivery vehicles, this study explains through a series of cytological and animal experiments how plasma exosomal circ_0001785 exerts its mechanism of action through the miR-513a-p/TGFBR3 ceRNA network pathway to attenuate ECs damage and delay the development of AS. In conclusion, this study provides new insights into the mechanism of action of circ_0001785 in the development of AS.

## Material and methods

### Patients

This study was approved by the Ethics Committee of the Second Affiliated Hospital of Harbin Medical University (approval number: KY2022-072). It is in accordance with the principles in the Declaration of Helsinki. In this study, we studied data from 31 patients with coronary heart disease and 24 patients without coronary heart disease treated at the Department of Cardiovascular Medicine, Second Affiliated Hospital of Harbin Medical University. All patients who participated in the study provided informed consent. The diagnostic criteria for coronary heart disease were reported according to the “Nomenclature and Diagnostic Criteria for Ischemic Heart Disease” developed by the Joint Working Group on Clinical Nomenclature Criteria of the International Society and Association of Cardiology and the World Health Organization (WHO). Patients with any chronic disease (including cancer, heart failure, diabetes mellitus, cirrhosis, and renal failure), connective tissue disease, inflammatory arthritis, active infection, or other inflammatory disease were excluded. Patient demographics are shown in Additional file 2: Table S1, S2.

### Animal models

The Animal Care and Use Committee of the Second Affiliated Hospital of Harbin Medical University approved all animal research protocols (approval number: SYDW2022-048). All experimental procedures were in accordance with the recommendations of the European Ethics Committee (EEC) (2010/63/EU). We purchased male C57BL/6 mice (8 weeks old) from the Experimental Animal Center of the Second Affiliated Hospital of Harbin Medical University. The ApoE^−/−^mice were purchased from Beijing Viton Lever Laboratory Animal Technology Co. The animals were housed individually in ventilated cages in standard animal rooms (temperature: 24–25 °C; humidity: 55%; 12-h light, 12-h dark photoperiod). C57BL/6 mice were given a normal supplemental diet. ApoE^−/−^ mice were fed a high-fat diet (21% fat, 20% protein, 50% carbohydrate) for 12 weeks to induce atherosclerosis.

To assess whether circ_0001785 delays the development of atherosclerosis in mice through exosomal transduction communication, we randomly assigned ApoE^−/−^mice to 3 groups and administered tail vein injections after 4 weeks of high-fat feeding. The specific groups were: control (vein injection of 100 μl PBS), overexpression virus (vein injection of 100 μl overexpression circ_0001785 lentivirus, virus titer 1*10^8^ TU/mL), overexpression virus control group (tail vein injection of 100 μl overexpression circ_0001785 control lentivirus, virus titer 1*10^8^ TU/mL). After the lentivirus injection, these mice continued to be fed a high-fat diet for 8 weeks. After 8 weeks, we removed the aorta and heart after terminal anesthesia (1.5% isoflurane inhalation) and cervical dislocation euthanasia.

### Extraction of exosomes

We isolated and purified exosomes from circulating blood using differential centrifugation. In detail, whole blood was collected and left at room temperature (25 ± 1 °C) for 20 min. The serum was then separated to remove blood cells and debris by centrifugation at 3000 × rpm for 15 min at 4 °C (5 ml of venous blood was collected from the patient). Next, we dilute 1 ml of the serum sample with 10 ml of PBS. It was then centrifuged at 10,000 × g for 15 min at 4 °C. The supernatant was then transferred to an ultracentrifuge tube and centrifuged at 100,000 × g for 1 h at 4 °C to obtain a white or clear precipitate. For further purification, the precipitate was washed with PBS and the purified exosomes were obtained by centrifugation again at 100,000 × g for 1 h at 4 °C. Finally, the isolated exosomes were resuspended and collected in sterile PBS for subsequent studies (stored in a refrigerator at − 80 °C).

### Tracing of exosomes

First, according to the instruction, we mixed diluent C reagent with PKH67 dye in a ratio of 1:9 to configure the mixture. Then the different groups of exosomes were added to the mixture of PKH67 in the ratio of 1:100. After a full reaction in a light-proof centrifuge tube, the mixture was incubated for 10 min and centrifuged at 100,000 × g for 70 min, followed by removal of the supernatant. We then fully dissolved the precipitate with 100 µl of PBS. And 10 μl of each group of exosomes were extracted for protein concentration determination (BCA method). Based on the extracted protein concentration, the amount of exosomes in each group was calculated (15 μg/mL). Next, we placed cell crawls into 6-well plates. We first allowed the endothelial cells to grow to 40% fusion in the 6-well plates, and then co-cultured the exosomes and endothelial cells that had been stained with PKH67 for 24 h. We then removed the cell crawls and washed them 3 times with PBS. Then we added 4% paraformaldehyde to the 6-well plates and fixed them for 30 min at room temperature, and washed them 3 times with PBS. We then stained the nuclei with DAPI for 10 min and washed them 3 times with PBS after staining. Finally, we removed the cell crawls and sealed the slices to observe whether the exosomes were phagocytosed by endothelial cells by confocal fluorescence microscopy.

### Transmission electron microscopic

First, we removed 10 µl of exosomes and added them dropwise to a copper grid for 1 min to allow them to precipitate, followed by aspiration of the sediment with filter paper. Afterwards, we added 10 µl of uranyl acetate dropwise to the copper grid for 1 min and again aspirated the sediment using filter paper. Next, we dried the sample at room temperature for several minutes. Finally, electron microscopy imaging was performed with 100 kV.

### Particle size analysis

First, we use the standard to perform the instrument performance test, and after passing the test, we perform the exosome sample loading. We take out 30 μl of exosomes for particle size analysis, and information on the particle size and concentration of exosomes detected by the instrument can be obtained after the sample analysis is completed.

### Fluorescent labeling and nano-flow cytometry detection

First, we added 30 µL of exosomes to 20 µL y of antibodies labeled with different fluorescence (IgG, CD9, CD81) and mixed thoroughly while incubating for 30 min at 37 °C under light-protected conditions. Then, we added 1 mL of pre-chilled PBS, selected an ultracentrifuge rotor and centrifuged for 70 min at 110,000 × g at 4 °C. Subsequently, we removed the supernatant and resuspended it by adding 50 μL of pre-chilled 1 × PBS. We first tested the performance of the instrument with standards, and after passing the test, the exosome samples were assayed, and once the assay was completed, the results of the protein index determination of the NanoFCM instrument were obtained.

### Culture of HUVECs

First, we resuspended the thawed HUVECs by centrifugation and inoculated them into culture flasks. After adding 5 ml of complete medium (90% DMEM + 10% FBS) to the culture flasks, they were incubated in an incubator at 37 °C. Then we observed the adnexal growth of endothelial cells with a microscope and performed passaging when the cell density reached more than 80%. When cell passaging was completed, we resuspended the cells by centrifugation at a ratio of 1:2 and reinoculated them into new culture flasks. Finally, we inoculated the fourth generation HUVECsf into 6-well plates, 24-well plates and 96-well plates for subsequent experiments.

### Statistical analysis

We used SPSS software (IBM SPSS Statistics 26.0) and GraphPad Prism 9.4.1 for statistical analysis. We used the t-test or ANOVA in the software to compare data with a normal distribution and the Wilcoxon test to compare data with a non-normal distribution. Statistical significance was set at P < 0.05.

Further information on the experimental methods has been described in the Additional file 1.

## Results

### Effects of plasma exosomes on endothelial cells in patients with coronary artery disease

**Fig. 1:**
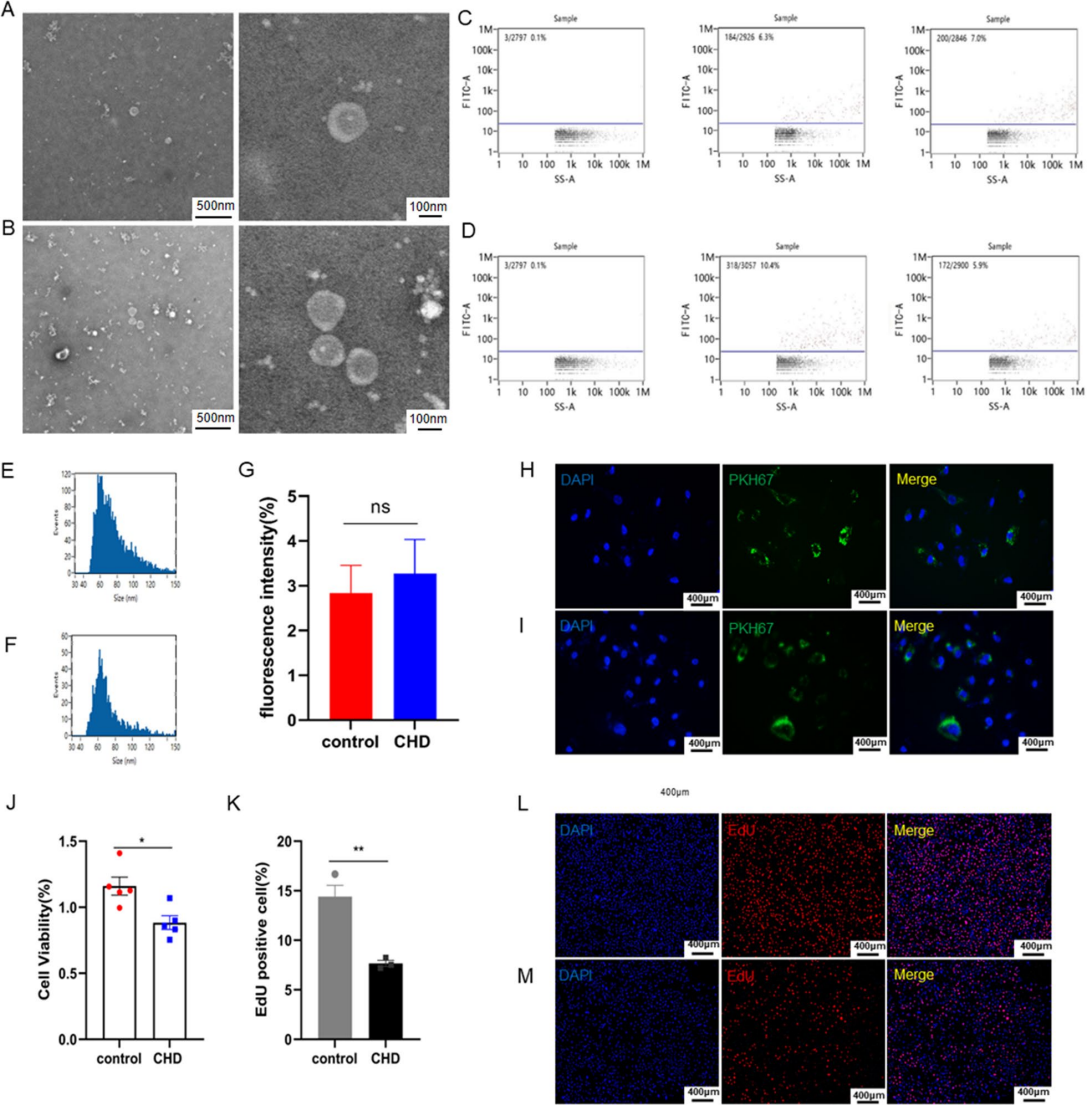
Identification and functional verification of circulating exosomes derived from CHD or non-CHD by differential centrifugation. **A**, **B** Ultrastructure of CHD-exosomes and non-CHD-exosomes showed approximately spherical morphology with TEM. **C**, **D**. Exosome-specific proteins markers were confirmed using nano-flow cytometry (nFCM). **E**, **F** Nanoparticle tracking analysis (NTA) showing the size distribution and average concentration of circulating exosomes in CHD and non-CHD. **G**, **I** PKH67-labeled exosomes were added to the HUVEC culture medium, and exosomes taken up by the HUVECs were visualized using confocal imaging. **J** CCK-8 of cell viability. **K**, **M** EdU of cell proliferation. Results are expressed as the mean ± SEM; student’s t-test or one-way ANOVA. *P < 0.05, **P < 0.01

First, we separated exosomes from patients with and without coronary artery disease by differential centrifugation. We observed spherical particles with diameters of 30–150 nm by transmission electron microscopy (TEM) (Fig. 1A, B), which is consistent with the size and morphology of exosomes. For the marker proteins CD9 and CD81 of exosomes, we examined them by nanoflow cytometry (nFCM) (Fig. 1C, D). In addition, we determined the size of exosomes by nanoparticle tracking analysis (NTA) technique (Fig. 1E, F). Interestingly, in electron microscopy of exosome samples from different diseases, we found that more exosomes were released in patients with coronary artery disease compared to those with non-coronary artery disease (Fig. 1A, B). To explore the effect of these exosomes on endothelial cells, we labeled exosomes from patients with and without coronary artery disease with PKH67 and cultured them into human umbilical vein endothelial cells (HUVECs). After 24 h of continuous labeling, we observed with confocal microscopy that the exosomes had been effectively internalized by HUVECs. But there was no significant change in the degree of internalization of endothelial cells by exosomes in patients with and without coronary artery disease (Fig. 1G, I). It is well known that endothelial cells are the first cells to respond to atherosclerosis. Therefore, to investigate the effect of exosomes on endothelial cells from patients with coronary artery disease, we subsequently tested the proliferative capacity and cell viability of endothelial cells (Fig. 1J, M). The results showed that plasma exosomes from patients with coronary artery disease attenuated the viability and proliferative capacity of endothelial cells.

### circ_0001785 Protection in the population

**Fig. 2:**
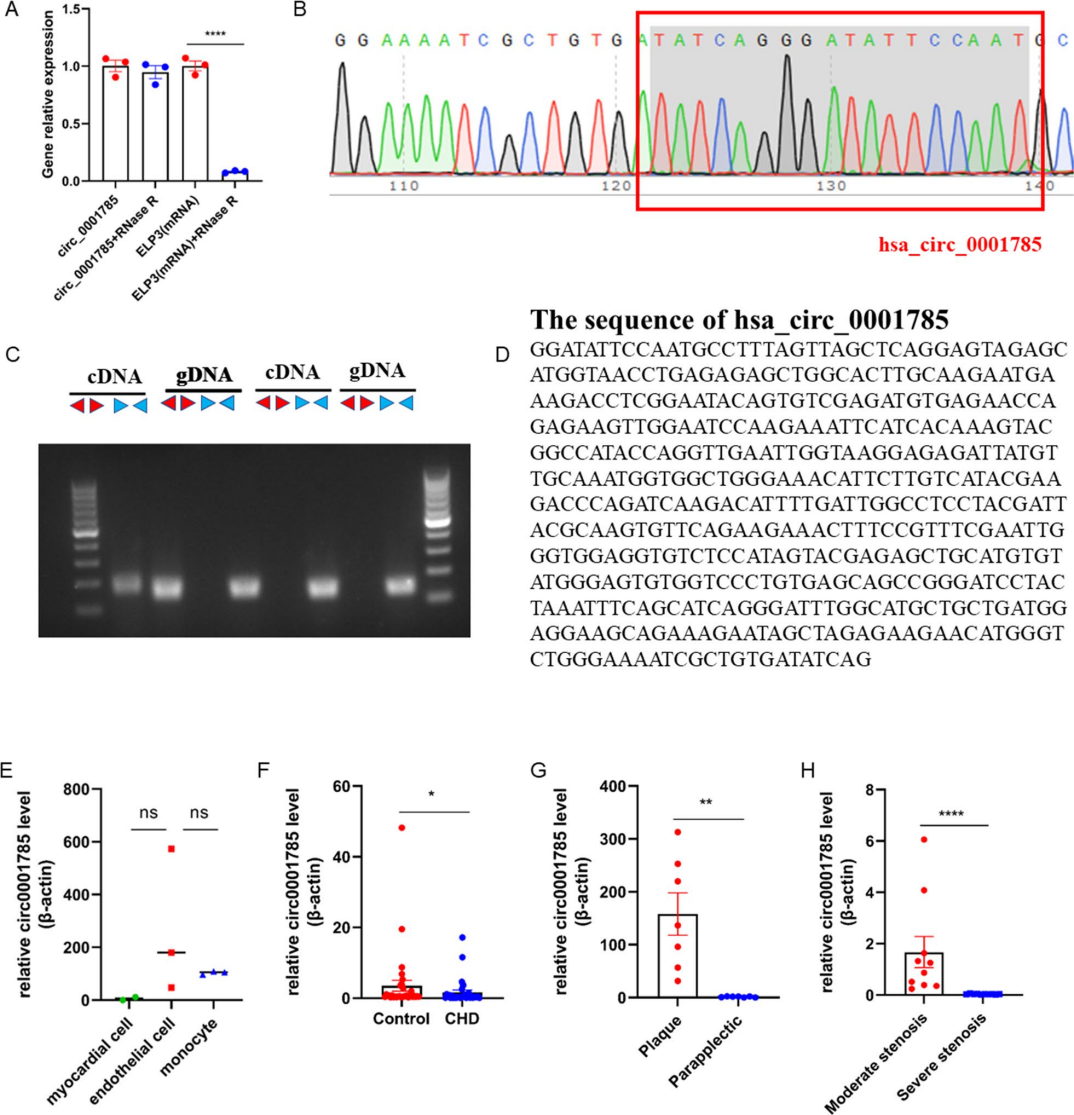
circ_0001785 protection in populations. **A** qRT-PCR analysis of circ_0001785 and linear ELP3 mRNA expression after RNase R digestion. **B** Sanger sequencing verification. **C** circ_0001785 and GAPDH expression in endothelial cell cDNA and gDNA (using divergent primers and polymerization primers, respectively). **D** The sequence of has_circ_0001785. **E** circ_0001785 had stable expression in monocytes. **F** Expression of circ_0001785 by qRT-PCR assay on CHD or non-CHD. **G** Expression of circ_0001785 in plaque tissue of patients with lower extremity atherosclerotic plaques. **H** Expression levels of circ_0001785 in patients with severe versus moderate coronary artery stenosis. Results are expressed as the mean ± SEM; student’s t-test or one-way ANOVA. *P < 0.05, **P < 0.01, **** P < 0.0001

CircRNA is difficult to be hydrolyzed by RNase R due to its unique properties of absence of 5′ and 3′ termini and poly A tail. To determine the structural features of circ_0001785, we first examined its tolerance to RNase R digestion. We treated total RNA extracted from endothelial cells with RNase R and tested the effect of RNase R treatment with its relative expression level to the host gene ELP3 linear RNA. The results showed that circ_0001785 was significantly tolerant to RNase R, further demonstrating the circular structure of circ_0001785 (Fig. 2A). To verify the reverse splice site of circ_0001785, we performed Sanger sequencing of the cDNA amplified by qRT-PCR for this circRNA Divergent primer. From the sequencing results, we could see that the circRNA contained the back-to-back splice site of the exon of ELP3 gene (Fig. 2B). In addition, we designed Convergent-specific primers for circ_0001785 and GAPDH, and Divergent-specific primers for circ_0001785 and GAPDH to amplify cDNA and gDNA of total RNA reversal of HUVECs, respectively.z Finally, it was found that circ_0001785 in cDNA could be detected in the cDNA, but not in the gDNA of HUVECs (Fig. 2C). We then investigated the effect of Exosome-circ_0001785 on atherosclerosis in patients with coronary artery disease. We first determined the cellular origin of circ_0001785. RNA was extracted from human cardiomyocytes, endothelial cells and monocytes, and then qRT-PCR experiments were performed. We found that circ_0001785 had stable expression in monocytes, and its expression was significantly higher than the other two groups. Therefore, we determined that circ_0001785 was mainly derived from monocytes (i.e., mainly from leukocytes) (Fig. 2E). Next, we extracted leukocytes from patients with coronary artery disease and detected the expression of circ_0001785 by qRT-PCR, and found that the expression level of circ_0001785 in leukocytes from patients with coronary artery disease was significantly lower than that in healthy control patients (Fig. 2F). In contrast, when we examined plaque tissue from patients with lower extremity atherosclerotic plaques, we found significantly more circ_0001785 in plaque tissue than in other tissues next to the plaque (Fig. 2G). Therefore, we suggest that circ_0001785 may be transferred to the site of injury via exosomes during plaque formation or endothelial cell injury. To further determine the relationship between circ_0001785 and patients with clinical coronary artery disease, we divided 20 patients into two groups according to the degree of coronary stenosis (grade III for 50%-75% and grade IV for 75%-100%), and the results showed that patients with severe coronary stenosis had significantly lower circ_0001785 levels than patients with moderate coronary stenosis levels (Fig. 2H).

### Functional science validation of ECs injury model

**Fig. 3:**
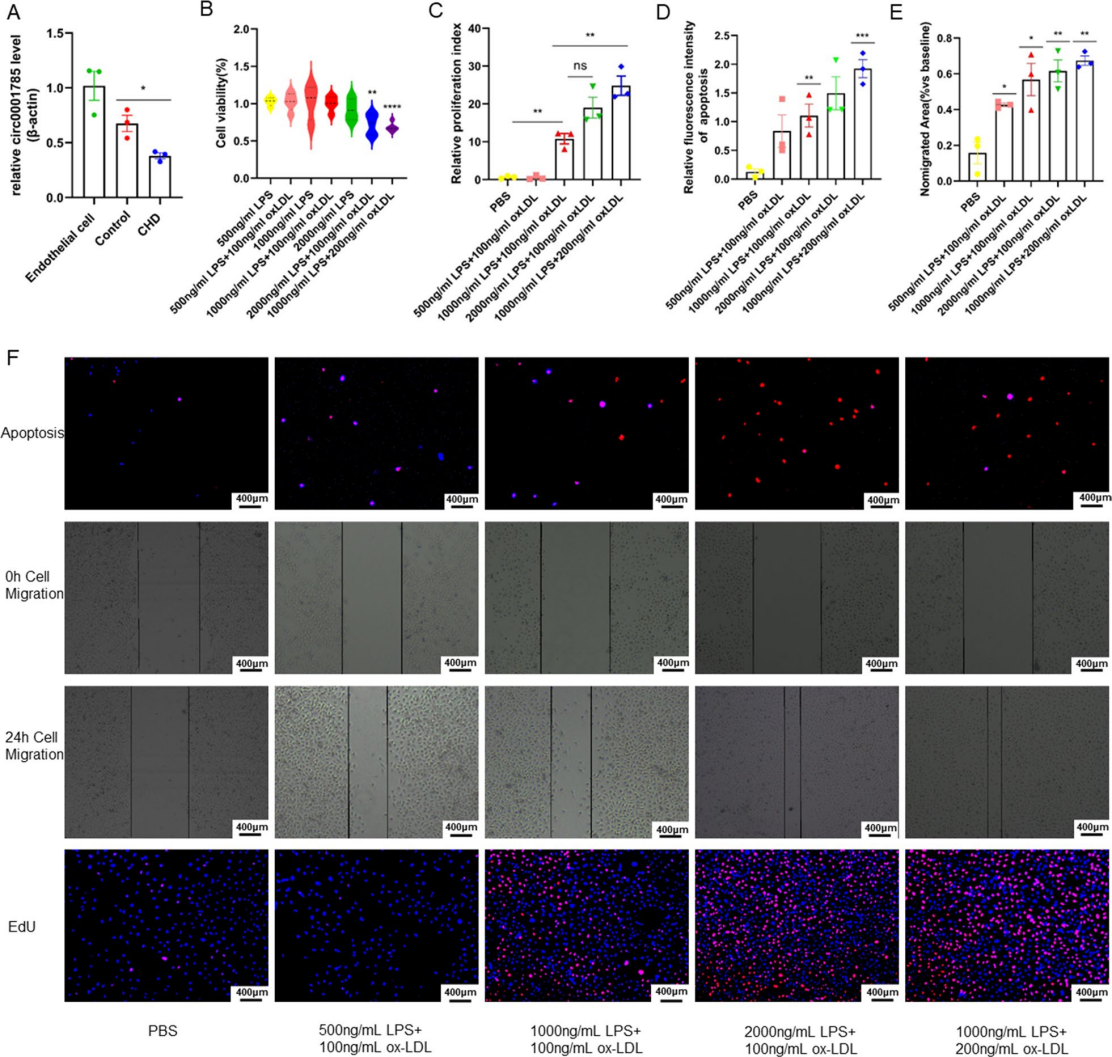
circ_0001785 induce proliferation of endothelial cells and inhibit apoptosis and migration of endothelial cells. **A** Expression of circ_0001785 after co-culture with endothelial cells. **B** Analysis of cell viability by CCK-8 assay **C** Analysis of cell proliferation by EdU assay **D** Analysis of apoptosis by PI + hoechst **E** Analysis of migration area by cell scratch assay. **F** Representative images of apoptosis, cell scratch, and proliferation. Results are expressed as the mean ± SEM; student’s t-test or one-way ANOVA. *P < 0.05, **P < 0.01, *** P < 0.001, **** P < 0.0001

First, we investigated the expression of circ_0001785 after co-culture of exosomes with endothelial cells and found that the expression of circ_0001785 was significantly lower in the CHD patient group (Fig. 3A). Therefore, we suggest that exosome-derived circ_0001785 may play an important role in coronary heart disease. To better mimic the environment in which human coronary heart disease develops, a new model of atherosclerotic endothelial cell injury was established in this study. We co-stimulated endothelial cells with ox-LDL combined with LPS, and confirmed the successful model establishment with functional experiments on endothelial cells, and again analyzed the biological functions of the model. In order to better simulate the developmental environment of human CHD, a novel model of AS with inflammatory infiltration was developed in this study. The model used ox-LDL combined with LPS to co-stimulate ECs. 1000 ng/ml LPS + 200 ng/ml oxLDL was found to be more toxic to ECs by CCK-8 assay versus 2000 ng/ml LPS + 100 ng/ml ox-LDL with statistical significance (Fig. 3B). Through functional validation of the ECs injury model, it was found that the degree of apoptosis was stronger and significant for 1000 ng/ml LPS + 100 ng/ml ox-LDL and 1000 ng/ml LPS + 200 ng/ml ox-LDL in the ECs apoptosis assay, but 2000 ng/ml LPS + 100 ng/ml ox-LDL had a less significant degree of apoptosis, which was considered here to be due to a stronger degree of cell death than apoptosis in 2000 ng/ml LPS + 100 ng/ml ox-LDL (Fig. 3D–F). In contrast, the wound healing assay and cell proliferation assay of ECs showed that the migration ability of ECs started to be enhanced when the concentration of ox-LDL and LPS reached 500 ng/ml LPS + 100 ng/ml ox-LDL (Figure 3E, F). A significant decrease in the proliferation of ECs occurs when ox-LDL and LPS concentrations exceed 1000 ng/ml LPS + 100 ng/ml ox-LDL (Fig. 3C–F). The present study provides a better quality model of ECs injury for simulating human AS.

### circ_0001785 exerts its functional through the ceRNA network axis of miR-513a-5p/TGFBR3

**Fig. 4:**
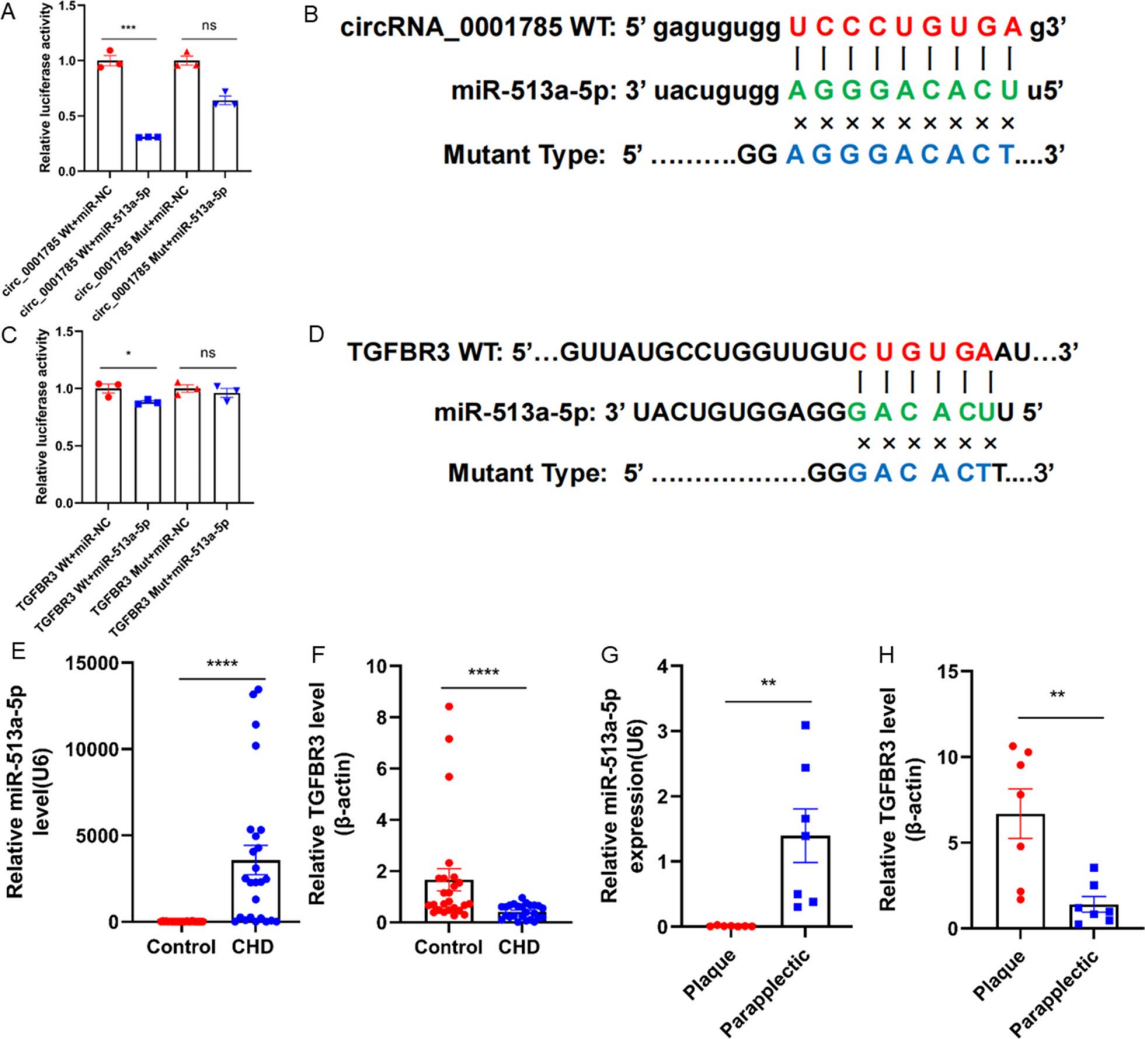
circ_0001785 exerts its functional through miR-513a-5p/TGFBR3ceRNA network axis. **A** Relative luciferase reporter activity of vectors carrying the luciferase gene and a fragment of circ_0001785 wild-type or mutant-type of miR-513a-5p binding sites after cotransfection with mimic NC or mimic miR-513a-5p. **B** The possible binding sites between circ_0001785 and miR-513a-5p. **C** Relative luciferase reporter activity of vectors carrying the luciferase gene and a fragment of TGFBR3 wild-type or mutant-type of miR-513a-5p binding sites after cotransfection with mimic NC or mimic miR-513a-5p. **D** The possible binding sites between TGFBR3 and miR-513a-5p. **E**, **F** qRT-PCR assay for the detection of miR-513a-5p and TGFBR3 expression in the blood of coronary heart disease and healthy patients. **G**, **H** qRT-PCR assay for the detection of miR-513a-5p and TGFBR3 expression in the plaque and paraplegic. Results are expressed as the mean ± SEM; student’s t-test or one-way ANOVA. *P < 0.05, **P < 0.01, *** P < 0.001, **** P < 0.0001

To identify the downstream target genes of circ_0001785, we first performed bioinformatics analysis. In a previous study, we identified the targeting pathway of circ_0001785/miR-513a-5p/TGFBR3 (10.3389/fcvm.2023.1070616). To validate the predicted targets, based on the possible binding sites between circ_0001785 and miR-513a-5p, we tested the binding ability of circ_0001785 to miR-513a-5p by a dual luciferase reporter gene assay in a base-complementary manner. We cotransformed miR-513a-5p mimics with a dual luciferase reporter gene plasmid containing circ_0001785 wild-type (Wt) and mutant (Mut) cDNA fragments into endothelial cells and found targeted mutations in the predicted binding site of circ_0001785 to miR-513a-5p. The results showed that overexpression of miR-513a-5p significantly reduced the Wt-Type circ_0001785 luciferase activity, but not the Mut-Type circ_0001785 luciferase activity (Fig. 4A, B). And the above results also indicated that circ_0001785 could directly bind to miR-513a-5p by base complementation. Similarly, we also verified the binding of miR-513a-5p and TGFBR3 (Fig. 4C, D). To further validate the expression of miR-513a-5p and TGFBR3, we extracted RNA from patients with coronary artery disease and healthy controls and performed qRT-PCR experiments for miR-513a-5p and TGFBR3, respectively, which showed increased expression of miR-513a-5p and significantly decreased expression of TGFBR3 in the blood of patients with coronary artery disease (Fig. 4E, F). Similarly, we examined plaque tissues from patients with lower extremity atherosclerotic plaques and found that the amount of miR-513a-5p was significantly less in plaque tissues than in other tissues next to the plaque, while the amount of TGFBR3 was more in plaque tissues than in other tissues next to the plaque (Fig. 4G, H).

### circ_0001785 promotes endothelial cell proliferation and inhibits apoptosis and migration via* miR-513a-5p/TGFBR3*

**Fig. 5:**
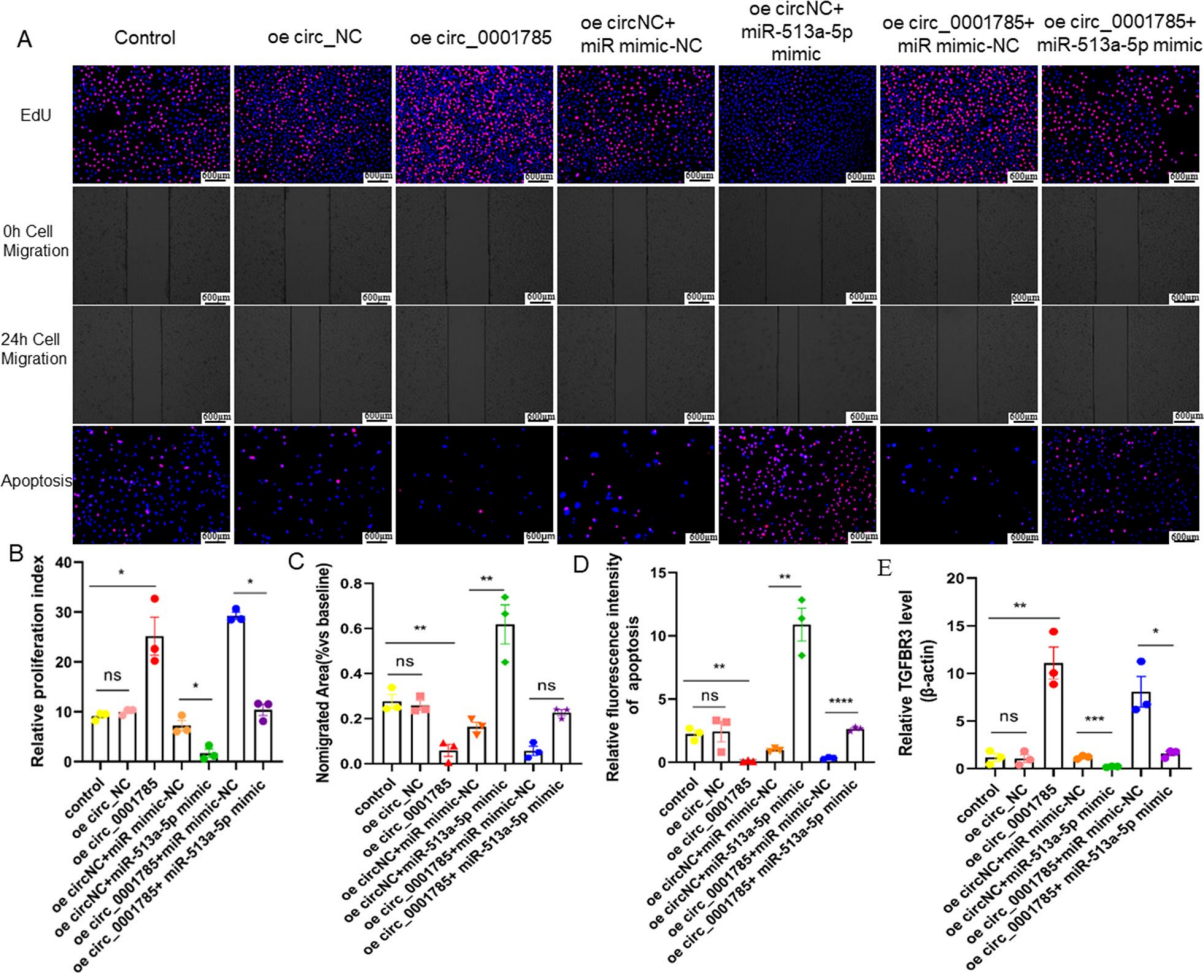
circ_0001785 promotes the proliferation and inhibits the apoptosis, and migration via miR-513a-5p/TGFBR3 in endothelial. **A** Representative images of apoptosis, cell scratch, and proliferation after 24 h of transfection into endothelial cells using overexpressed circ_0001785 or circ_NC. **B** Analysis of cell proliferation by EdU assay. **C** Analysis of migration area by cell scratch assay. **D** Analysis of apoptosis by PI + hoechst. **E** qRT-PCR assay for the detection of TGFBR3 expression after 24 h of transfection into endothelial cells using overexpressed circ_0001785 or circ_NC. Results are expressed as the mean ± SEM; student’s t-test or one-way ANOVA. *P < 0.05, **P < 0.01, *** P < 0.001, **** P < 0.0001

Through the above experiments, we have demonstrated that circ_0001785 can inhibit the apoptosis and migration of endothelial cells. And circ_0001785 can indirectly affect the expression of TGFBR3 through miR-513a-5p. To further explore whether circ_0001785 affects endothelial cell proliferation, migration and apoptosis through miR-513a-5p/TGFBR3 pathway. In this study, we cotransfected overexpressed circ_0001785 with miR-513a-5p mimics and miR-513a-5p NC, respectively, and validated them using cell functional assays. The specific groups were control group, oe circ_NCz group, oe circ_0001785 group, oe circ_NC + miR mimic-NC group, oe circNC + miR-513a-5p mimic group, oe circ_0001785 + miR mimic-NC group, oe circ_0001785 + miR-513a-5p mimic group. The results showed that overexpression of miR-513a-5p somewhat attenuated the proliferation promoting effect caused by circ_0001785 overexpression (Fig. 5A, B), as well as the inhibitory effect on apoptosis (Fig. 5C), and endothelial cell migration (Fig. 5D). In contrast, the addition of miR-513a-5p NC plasmid had no significant effect on the function of endothelial cells. We also examined the expression level of TGFBR3 by qRT-PCR and found that the expression level of TGFBR3 was significantly increased in endothelial cells overexpressing circ_0001785, while the addition of miR-513a-5p mimics was able to antagonize this increase in expression, resulting in a decrease in TGFBR3 expression. In contrast, the addition of miR-513a-5p NC plasmid did not significantly change the expression of TGFBR3 in the endothelium (Fig. 5E). The above experiments suggest that circ_0001785 can indirectly regulate the expression of TGFBR3 by inhibiting miR-513a-5p, thus exerting its promotional effect on endothelial cell proliferation and its inhibitory effect on endothelial cell apoptosis and migration.

### Construction of an endothelial injury model of atherosclerosis in mice

**Fig. 6:**
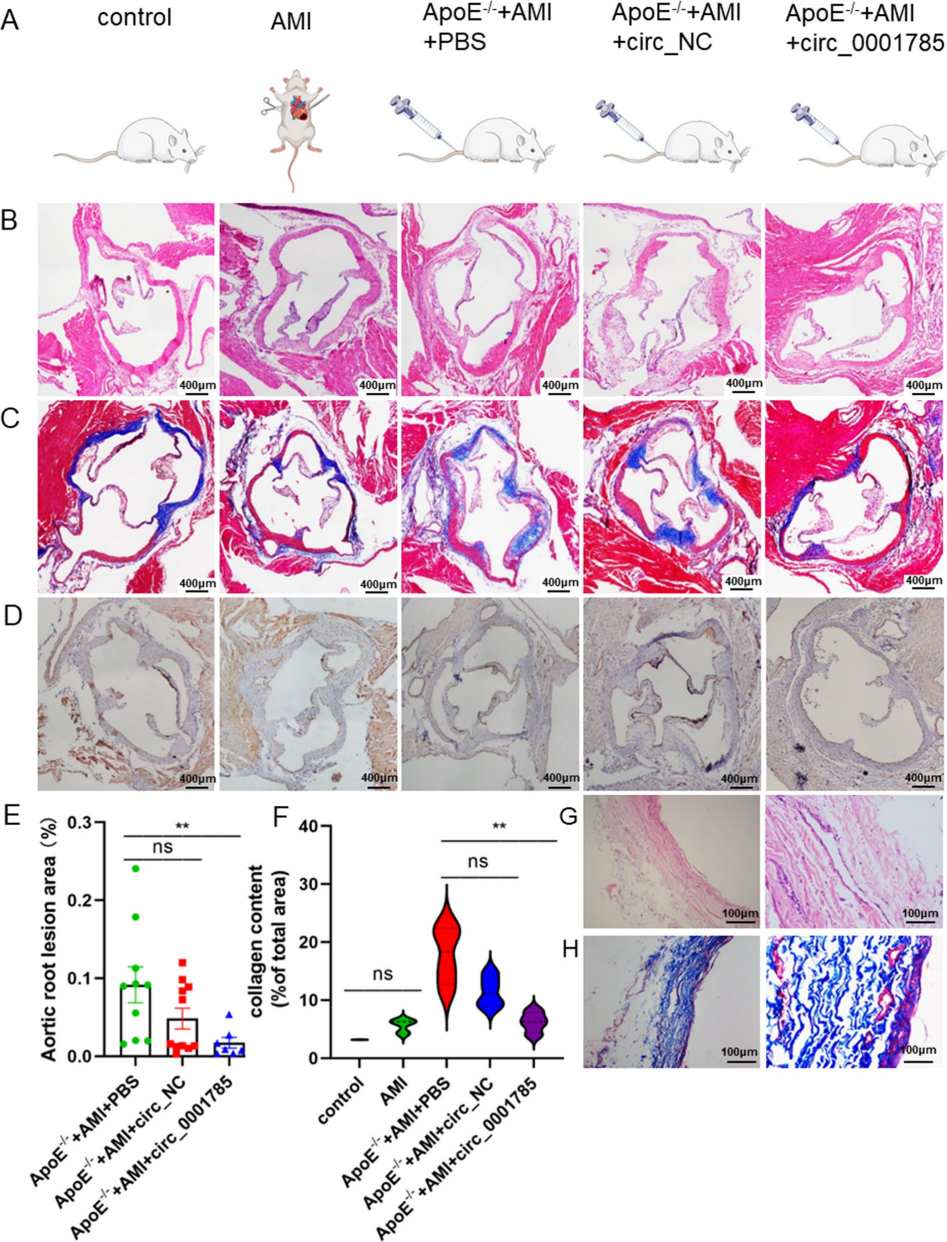
Establishment of an in vivo model of endothelial injury in atherosclerosis **A** Mice were divided into 5 groups. The tail vein of mice was treated with circRNA. **B** HE-stained images of atherosclerotic lesions and aortic root lesion areas **C** Masson stained images of paraffin sections **D** Immunohistochemical staining of endothelial CD31 at the aortic root lesion in mice. **E** HE staining of aortic root lesion area **F** Massonr staining of collagen area **G**, **H** HE and Masson staining of plaque tissue in patients with lower extremity atherosclerosis. Results are expressed as mean ± SEM; Student’s t test or one-way ANOVA. **P < 0.01

As with the cellular model, we established a mouse model with both atherosclerotic plaque and inflammatory infiltration. Briefly, we fed mice with a high-fat diet and then performed a uniform left anterior descending branch ligation. However, the aim was not to infarct, but to cause an inflammatory infiltrate around the ligation line at the time of ligation, thus further approximating the nature of human vulnerable plaques. Interestingly, we found that after ligation of the left anterior descending branch in mice, inflammation around the ligature line could spread directly to the aortic sinus, leading to inflammatory infiltration at the aortic sinus plaque, which facilitated our further observation. The specific groups were: control, AMI, ApoE^−/−^ + AMI, ApoE^−/−^ + AMI + PBS, ApoE^−/−^ + AMI + LV-oe circ_0001785, ApoE^−/−^ + AMI + LV-oe circNC. where ApoE^−/−^ mice were given high-fat feeding for 10 weeks before left anterior descending branch ligation (Fig. 6A). ApoE^−/−^ + AMI mice were more prone to endothelial cell detachment and inflammatory infiltration with plaque enlargement compared with the control and AMI groups (Fig. 6B, D, E). Also, mice in the model group had increased atherosclerosis and were prone to increased collagen content and thinning of the fibrous cap (Fig. 6C, F). To observe the consistency of the mouse model with human plaque tissue, we removed plaque tissue from patients with lower extremity atherosclerosis from the clinic and then performed HE and Masson staining, and we found that both inflammatory infiltration and collagen fibers were significantly increased in plaque tissue from patients with lower extremity atherosclerosis, consistent with the lesion characteristics of atherosclerotic vulnerable plaques in the mouse model (Fig. 6G, H).

### Overexpression of circ_0001785 leads to reduced endothelial injury and enhanced left ventricular diastolic function in mice

**Fig. 7:**
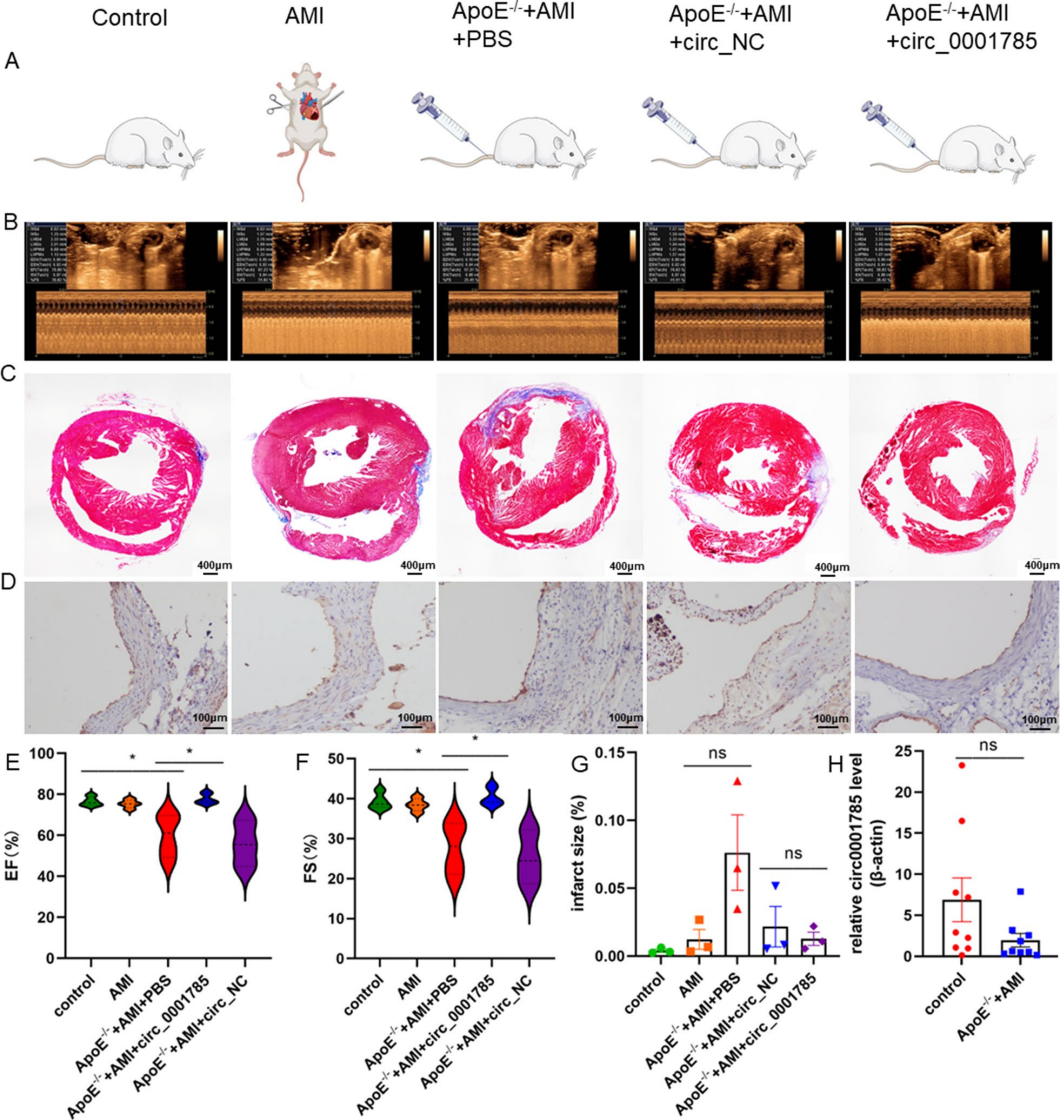
Overexpression of circ_0001785 leads to reduced endothelial injury and enhanced left ventricular diastolic function in mice **A** Mice were divided into 5 groups. ApoE^−/−^ mice were injected with circRNA in the tail vein. **B** Echocardiographic testing to analyze cardiac function after the onset of AMI in each group (left). **C** HE-stained images of atherosclerotic lesions and aortic root lesion areas. **D** Immunohistochemical staining of endothelial CD31 at the aortic root lesion in mice. **E** Percentage of left ventricular ejection fraction (EF). **F** Percentage of short-axis shortening (FS) of the left ventricle. **G** Area of HE staining of aortic root lesions. **H** qRT-PCR detection of circ_0001785 expression in control and ApoE.^−/−^ + AMI mice. Results are expressed as mean ± SEM; Student's t-test or one-way ANOVA. *P < 0.05

In the present experiment, we induced AMI in mice with ligation of the left anterior descending branch of the coronary artery (LAD) and found inflammatory infiltration at the aortic sinus. Therefore, we investigated the effect of circ_0001785 overexpression on cardiac function after AMI by focusing on echocardiography. Both EF and FS were improved after overexpression of circ_0001785 treatment compared with the PBS group or circ_NC group, suggesting that overexpression of circ_0001785 slowed left ventricular (LV) dysfunction after myocardial infarction (Fig. 7B, E–F). Using Masson staining, we observed a significant reduction in infarct size in circ_0001785 lentivirus-injected mice compared with overexpression of circ_NC and PBS controls (Fig. 7C–G). Since human vulnerable plaques are characterized by intraplaque neovascularization and intraplaque hemorrhage, we tested whether these features are also present in ApoE^−/−^ + AMI mice. We found that neovascularization was observed in the plaques of mice in the ApoE^−/−^ + AMI + PBS group, but no significant neovascularization was observed in mice in the overexpression circ_0001785 group (Fig. 7D). To further verify the role played by circ_0001785 in the mouse model, we verified the expression of circ_0001785 in the blood of healthy and model mice using qRT-PCR assay found that the expression of circ_0001785 in the blood of mice in the model group was significantly reduced.

## Discussion

Atherosclerotic cardiovascular disease (ASCVD) is a global health problem, which leads to many adverse heart accidents and cardiovascular deaths [22]. The gold standards for treating atherosclerosis are to prevent cardiovascular accidents by artificially controlling risk factors, such as smoking, hypertension, and hyperlipidemia, and to recover blood flow through surgery [23, 24]. However, more relevant research and strategy need to be proposed because of the limitations regarding the treatment of cardiovascular disease. Biological markers associated with ASCVD have shown potential in the treatment of ASCVD but need to be further explored [25]. In addition, the introduction of extracellular vesicle (EV) biology in the field of regenerative medicine has led to a growing interest in researchers to understand cardiovascular diseases based on the intercellular communication mechanisms of EV. It has been demonstrated that EVs play a role in all stages of atherosclerotic disease [26]. For example, high levels of circulating EVs are associated with pathological states such as dyslipidemia, diabetes mellitus, and inflammatory diseases, suggesting that EVs are involved in many of the processes that cause atherogenesis, including endothelial dysfunction, apoptosis, vascular remodeling, and immune responses [27, 28]. Although many studies demonstrate the role of EVs in atherosclerotic plaque stabilization, the effect of circulating exosomes in plaque formation remains unknown [29–31]. Based on these, circulating exosomes may be potential candidates that can be studied in atherosclerosis therapeutic strategies.

In this study, we investigated the effect of circRNA in exosomes on atherosclerosis. First, we extracted exosomes from plasma of patients with coronary heart disease and then co-cultured them with endothelial cells. As a result, we found that exosomes from plasma of coronary heart disease patients decreased the viability and proliferation of endothelial cells. Meanwhile, exosome-derived circ_0001785 protected endothelial cells from damage and reversed atherosclerosis-induced endothelial cell dysfunction. This effect was less pronounced with increasing coronary artery stenosis. We then validated that circ_0001785 protects endothelial cells by promoting their proliferation and inhibiting their apoptosis and migration through the ceRNA network mechanism of miR-513a-5p/TGFBR3. To further validate the role of circ_0001785, we injected circ_0001785 into mice to verify the protective effect of circ_0001785 on endothelial cells and its ability to retard the development of atherosclerosis in in vivo experiments. Thus, this study contributes to further understanding of new therapeutic strategies for atherosclerotic plaques.

The previous report has shown that circ_0001785 can inhibit the proliferation, migration, and invasion of breast cancer cells, either in vitro or in vivo, by up-regulating SOCS3 through sponging miR-942 [32]. miR-513a-5p can improve the apoptosis mediated by TNF-a and LPS through downregulating XIAP in endothelials [33]. In addition, TFGFBR3 also plays an important role in protecting myocardial cells after myocardial infarction [34]. It is worth noting that exosomes can serve as an essential mediator for the delivery of these RNAs. Exosomes originating from a variety of cells are intracellular membrane-bound vesicles with a size of 30–150 nm in diameter and can transfer their bioactive molecules between cells [35]. The protective effect of exosomes on the cardiovascular system has been well demonstrated in previous reports [36–38]. For example, exosomes produced during myocardial ischemia can mediate the prevention and treatment of cell transplantation [39]. These previous results shed light on our study and provide a strong theoretical basis for our experiments. Here, we verified the presence of protective circRNAs in exosomes which can delay the development of atherosclerosis through the analysis of the ceRNA network axis of circ_0001785/miR-513a-5p/TGFBR3.

The limitation of this study is that we did not consider the difference between the circRNA from patients with acute ST-segment elevation myocardial infarction and acute non-ST-segment elevation myocardial infarction, whereas atherosclerosis is prone to affect acute non-ST-segment elevation myocardial infarction. This question will be further investigated in the next study. In addition, mouse models used in this study may not be able to fully emulate the complex condition of myocardial infarction in humans. Therefore, more established models of atherosclerotic plaque rupture in mice need to be explored with the contribution of researchers. Finally, due to the limitations of experimental techniques, we did not further study the reasons for the reduced expression of circ_0001785 in plasma and the increased expression of circ_0001785 in plaque tissue, and these questions need to be further addressed in future studies.

In summary, we demonstrate that circ_0001785 protects against endothelial cell injury by sponging miR-513a-5p to upregulate TGFBR3, thereby slowing the onset of atherosclerosis, which improves our understanding of the endogenous mechanisms of atherosclerotic plaque formation. In the future, more studies on clinically relevant large animal models are necessary to simulate clinical situations before making inferences about the potential impact of our findings on humans.

## Conclusion

**Fig. 8:**
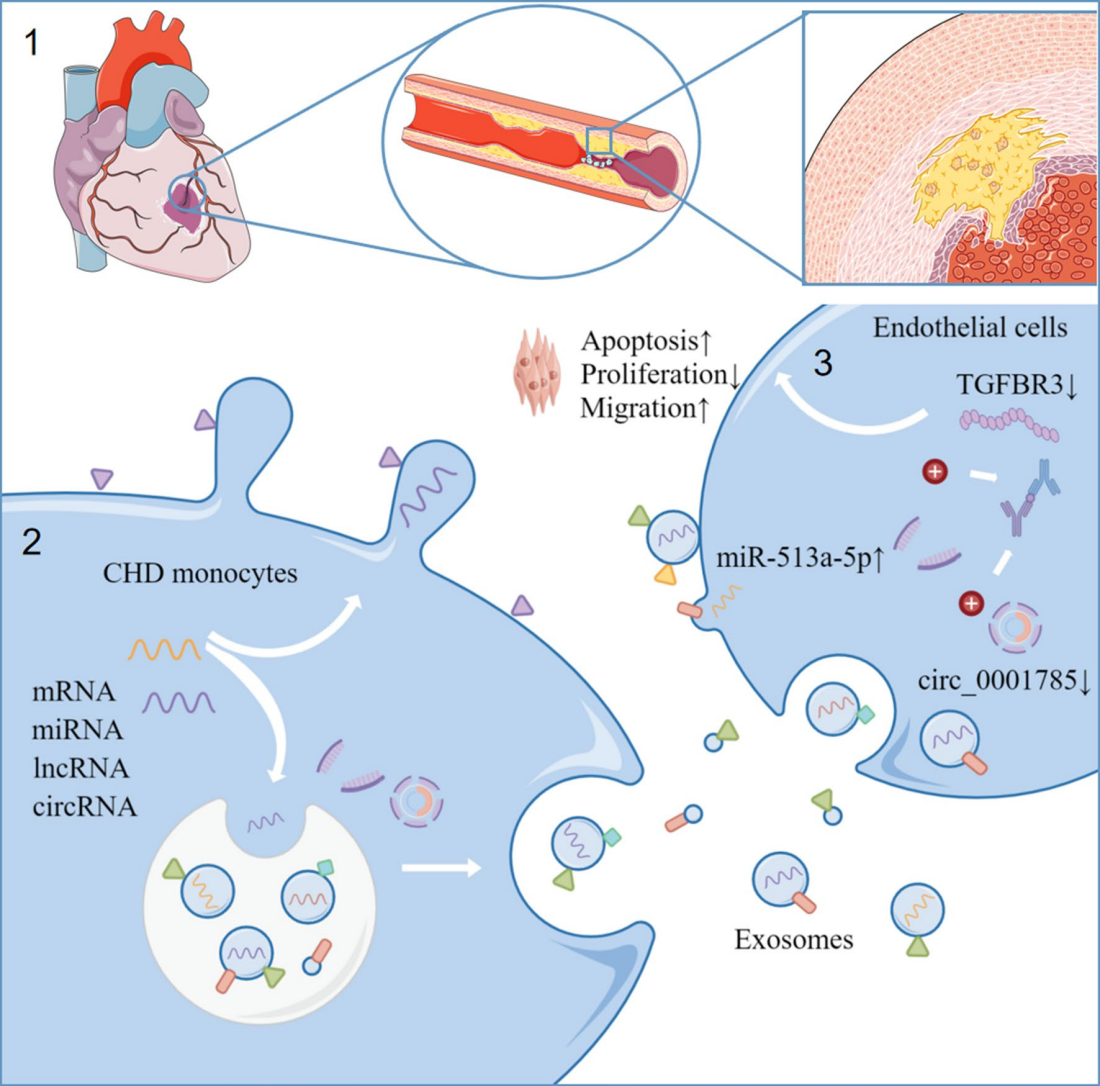
Flow chart. In this experiment, we demonstrated that the plasma exosome circ_0001785 functions as a ceRNA to regulate the downstream expression of TGFBR3 by "sponging" miR-513a-5p. Through this pathway, circ_0001785 promoted the proliferation and cell viability of ECs, and inhibited the apoptosis and migration of ECs, resulting in a reduction of plaque size and improved cardiac function in mice with AS, thus affecting the development and progression of AS

In conclusion, this paper shows that circ_0001785 in exosomes acts as a ceRNA to regulate downstream TGFBR3 expression by "sponging" miR-513a-5p. Through this pathway, circ_0001785 achieves the promotion of endothelial cell proliferation and the inhibition of apoptosis and migration. Thus, it influences the development of atherosclerotic plaques (Fig. 8).

### Supplementary Information

**Additional file 1.** Basic clinical patient information.

**Additional file 2.** The primer sequence of qRT-PCR test. **Table S1**. Basic clinical patient information. **Table S2**. The primer sequence of qRT-PCR test.

## Data Availability

All data generated or analyzed during this study have been included in the article and the Additional information.

## Abbreviations

ceRNA: Competitive endogenous RNA
HUVECs: Human Umbilical Vein Endothelial Cells
qRT-PCR: Real-time quantitative reverse-transcription PCR
ECs: Endothelial cells
AS: Atherosclerosis
CHD: Coronary atherosclerotic heart disease
DEGs: Differentially expressed genes
