# Supplementary figures and images for: Exosome-derived circ_0001785 delays atherogenesis through the ceRNA network mechanism of miR-513a-5p/TGFBR3
Source: J Nanobiotechnology. 2023 Oct 4;21:362. doi: 10.1186/s12951-023-02076-x (PMC10548746; doi:10.1186/s12951-023-02076-x)

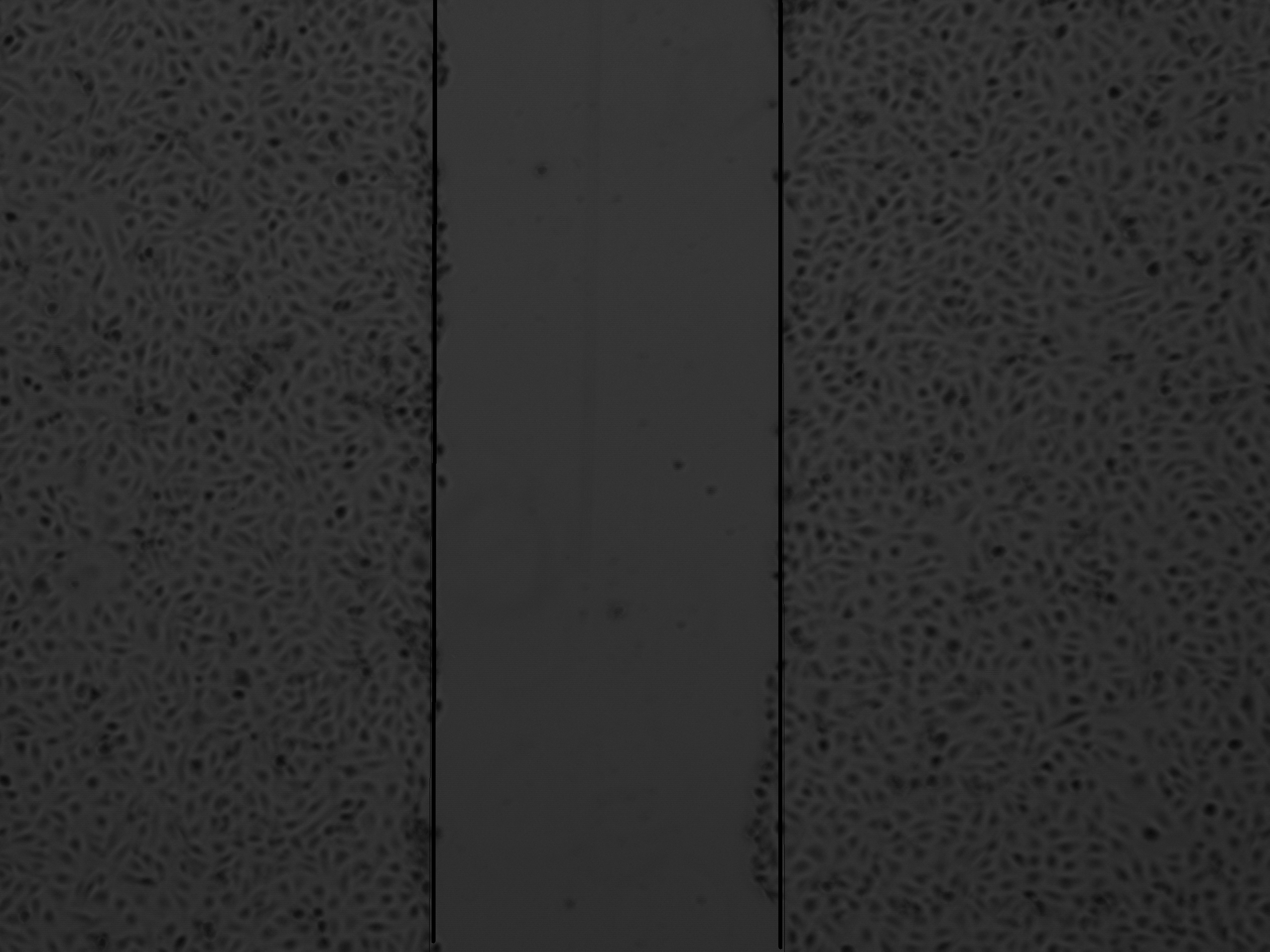

Supplement: Supplementary file 1 — Additional file 1. Basic clinical patient information. [file 12951_2023_2076_MOESM1_ESM.zip › con-1-0h.png]

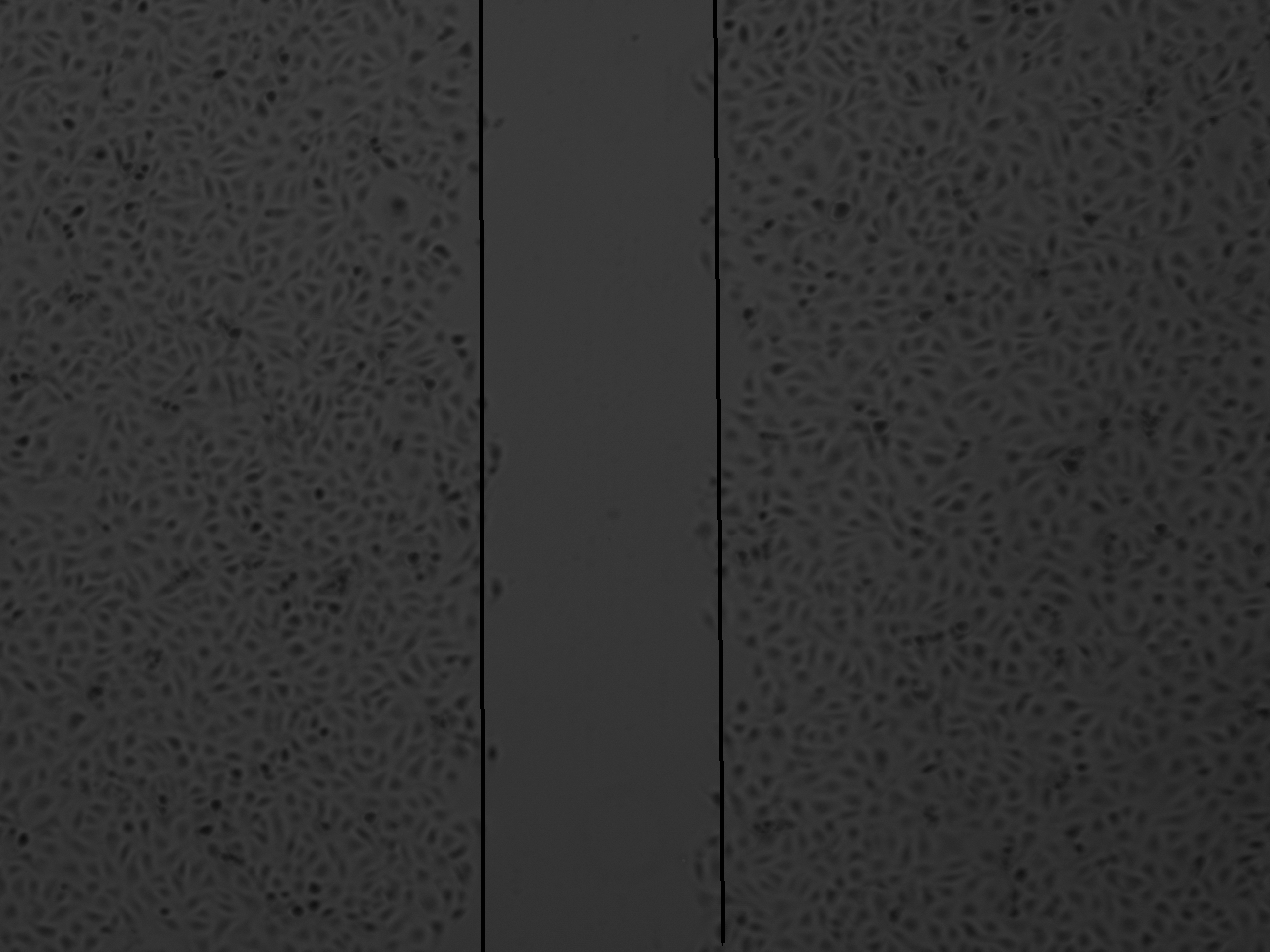

Supplement: Supplementary file 1 — Additional file 1. Basic clinical patient information. [file 12951_2023_2076_MOESM1_ESM.zip › con-1-24.png]

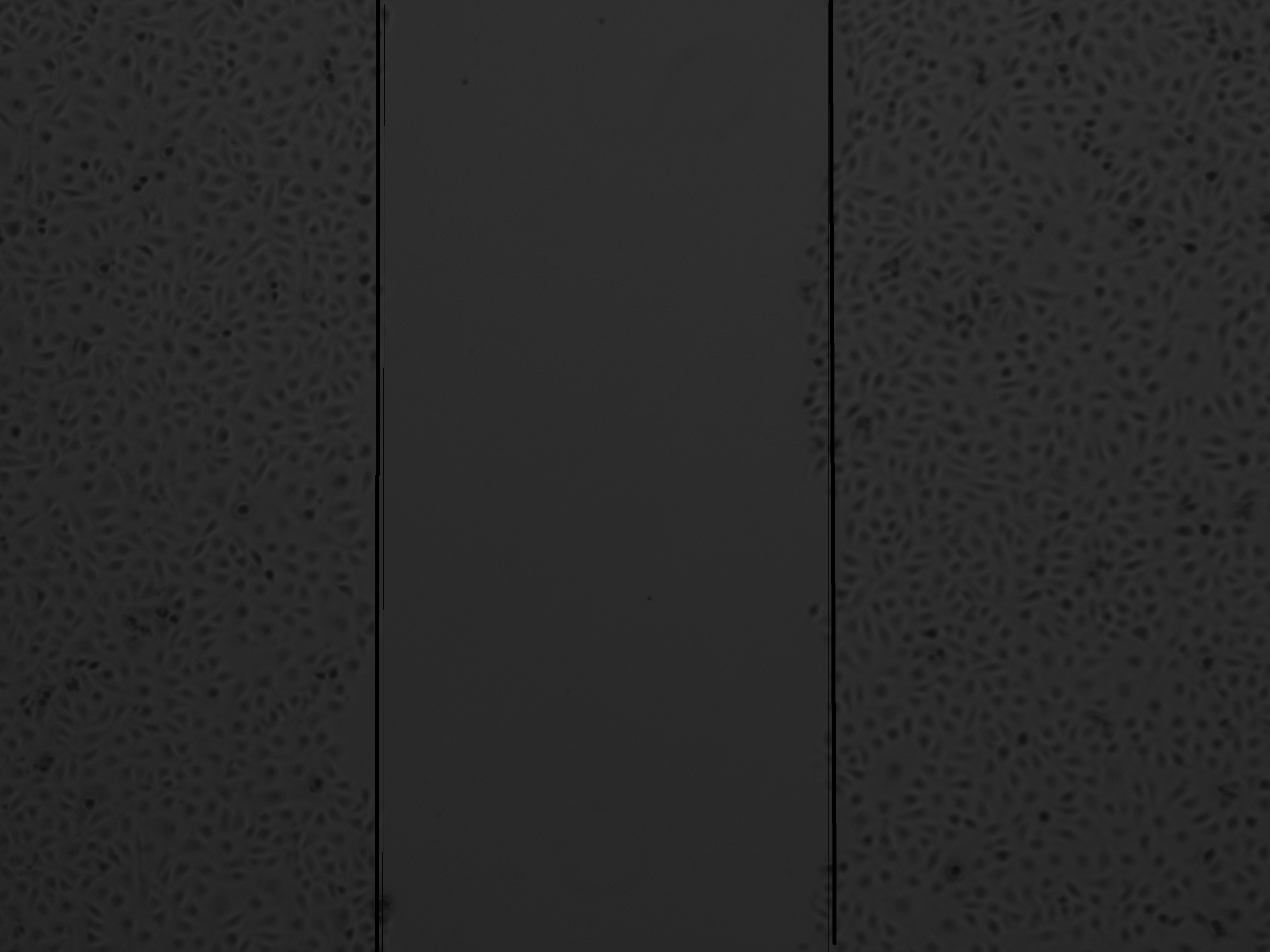

Supplement: Supplementary file 1 — Additional file 1. Basic clinical patient information. [file 12951_2023_2076_MOESM1_ESM.zip › oe circ NC+miR-513a-5p.png]

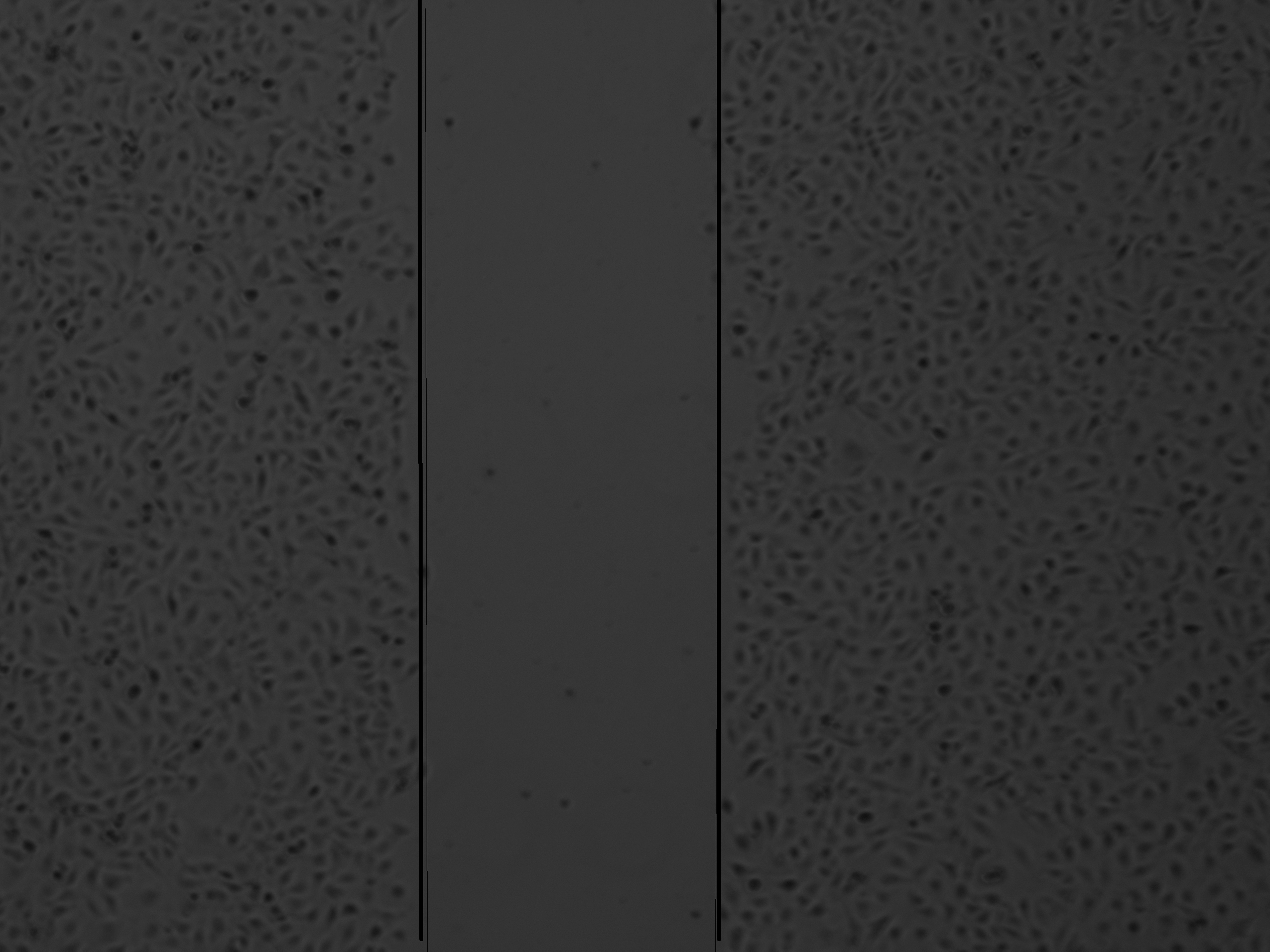

Supplement: Supplementary file 1 — Additional file 1. Basic clinical patient information. [file 12951_2023_2076_MOESM1_ESM.zip › oe circ_0001785 24h.png]

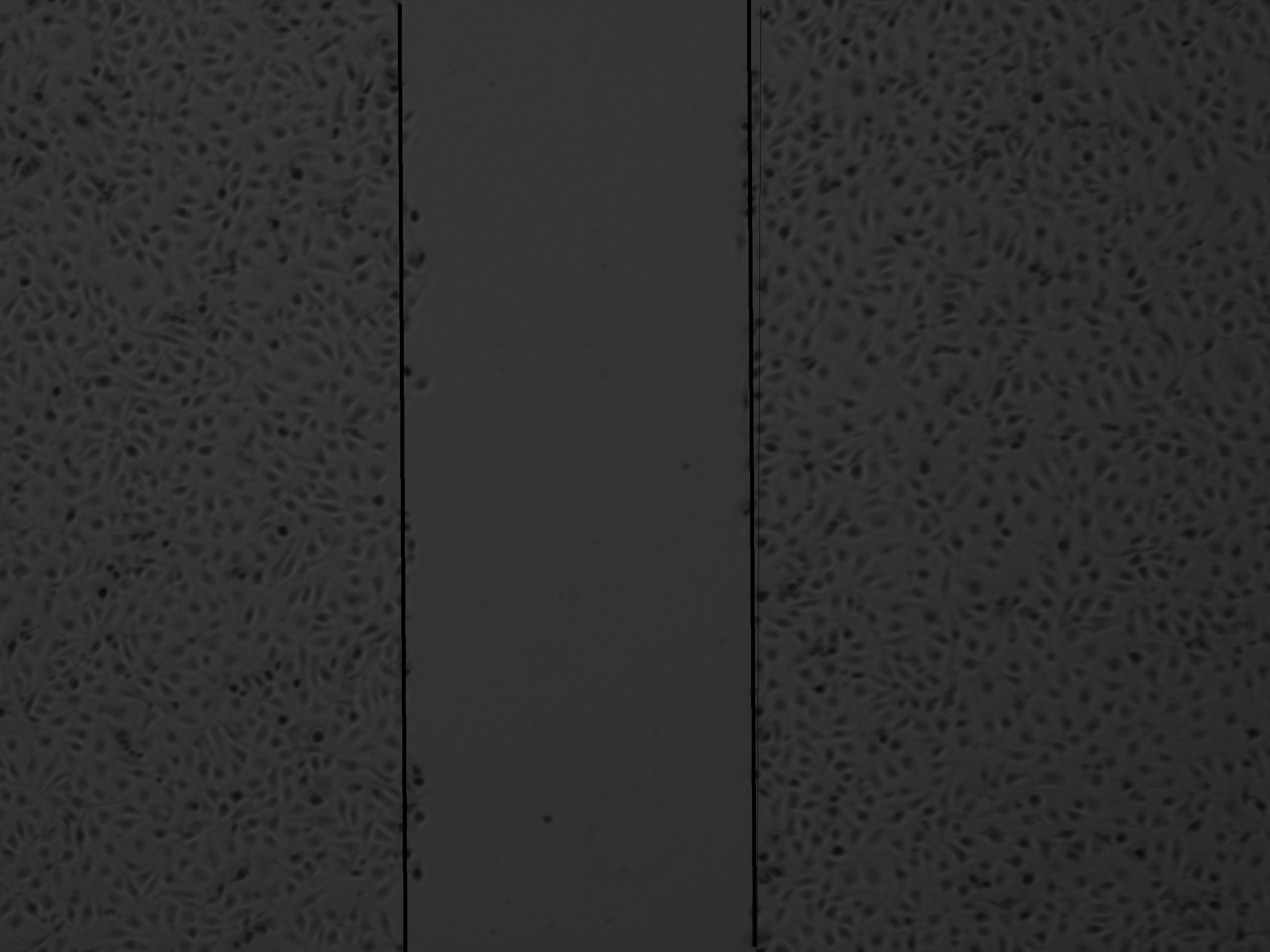

Supplement: Supplementary file 1 — Additional file 1. Basic clinical patient information. [file 12951_2023_2076_MOESM1_ESM.zip › oe circ_0001785.png]

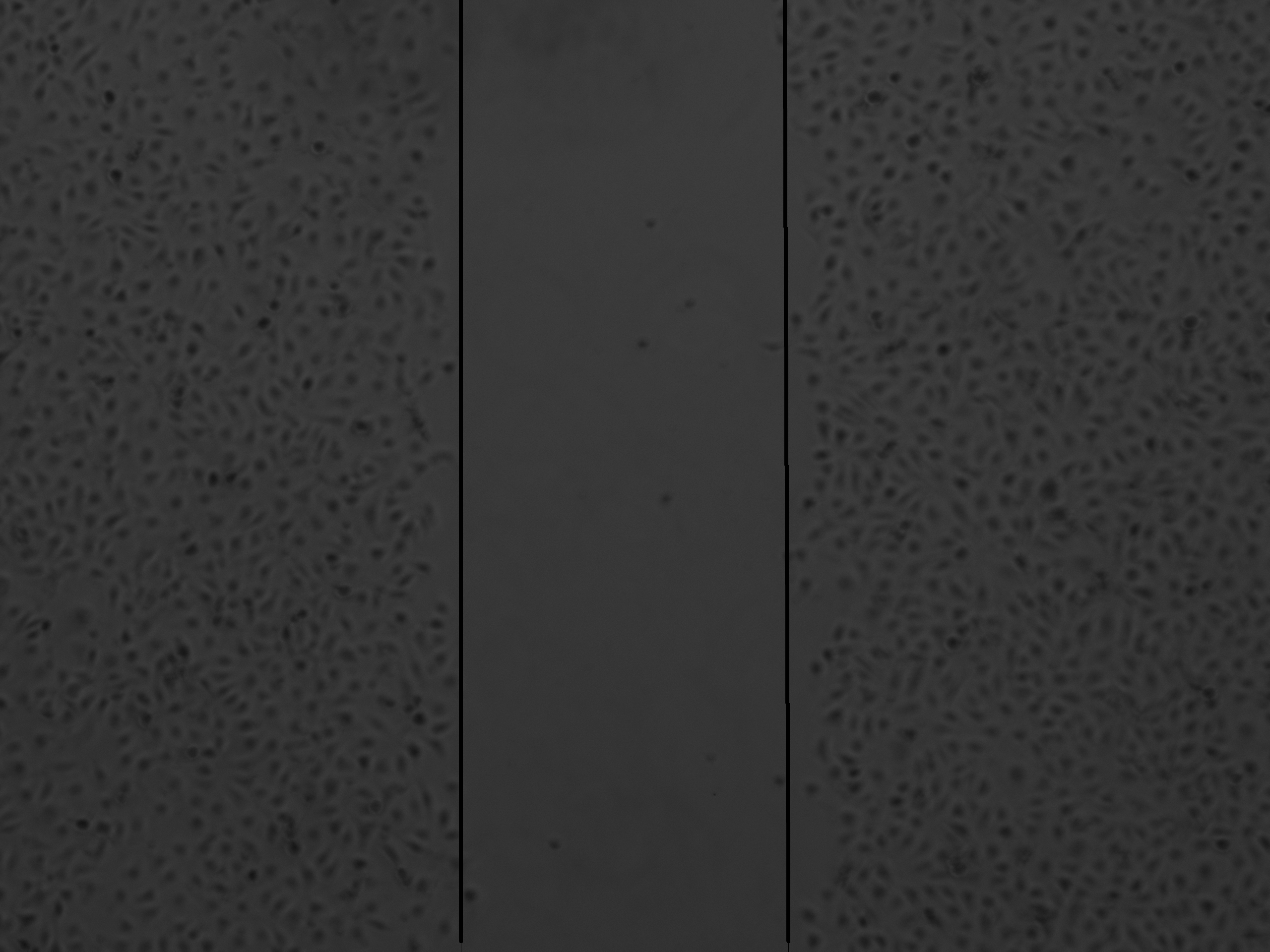

Supplement: Supplementary file 1 — Additional file 1. Basic clinical patient information. [file 12951_2023_2076_MOESM1_ESM.zip › oe circ_0001785+miR NC 24h.png]

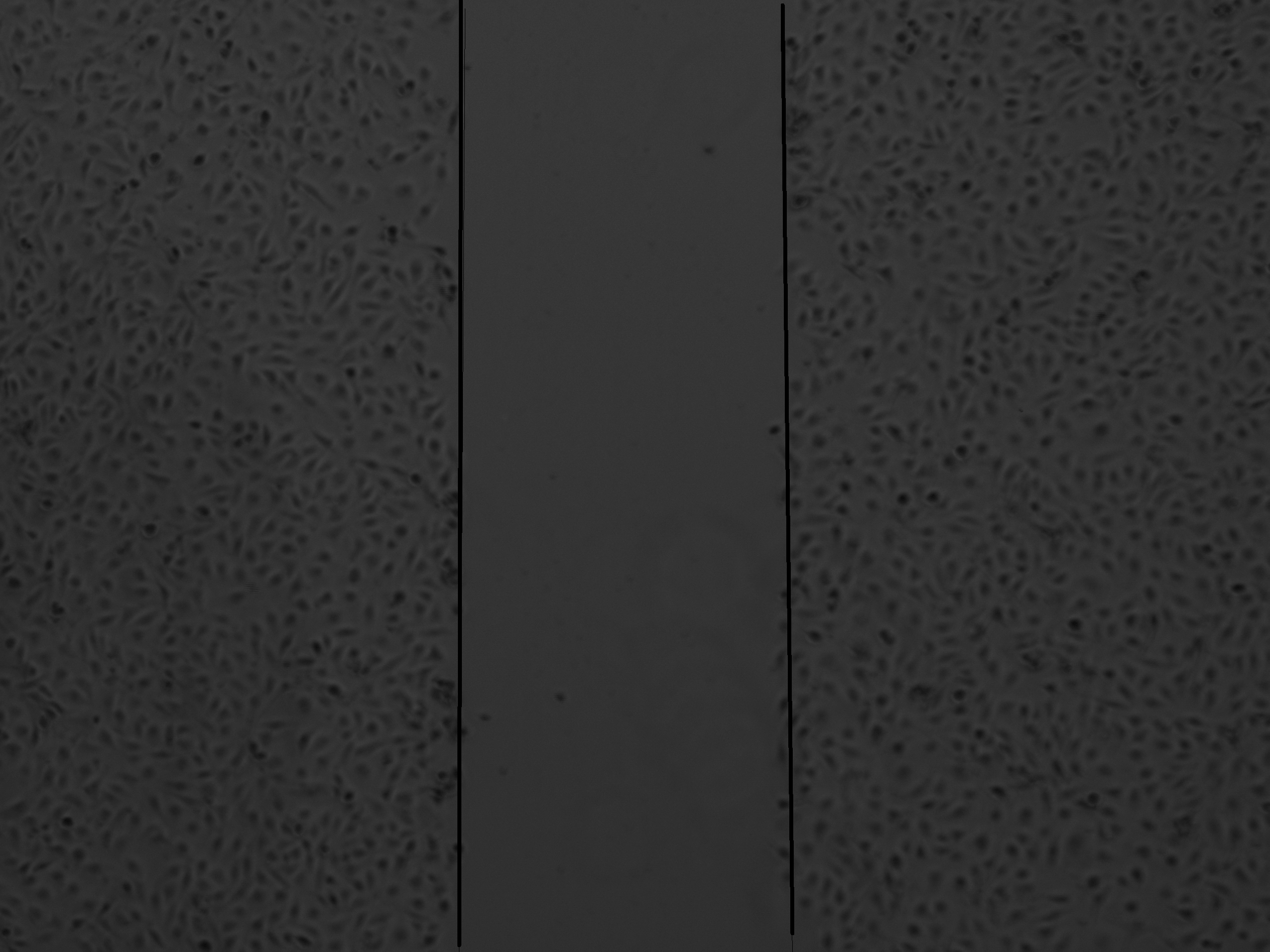

Supplement: Supplementary file 1 — Additional file 1. Basic clinical patient information. [file 12951_2023_2076_MOESM1_ESM.zip › oe circ_0001785+miR NC.png]

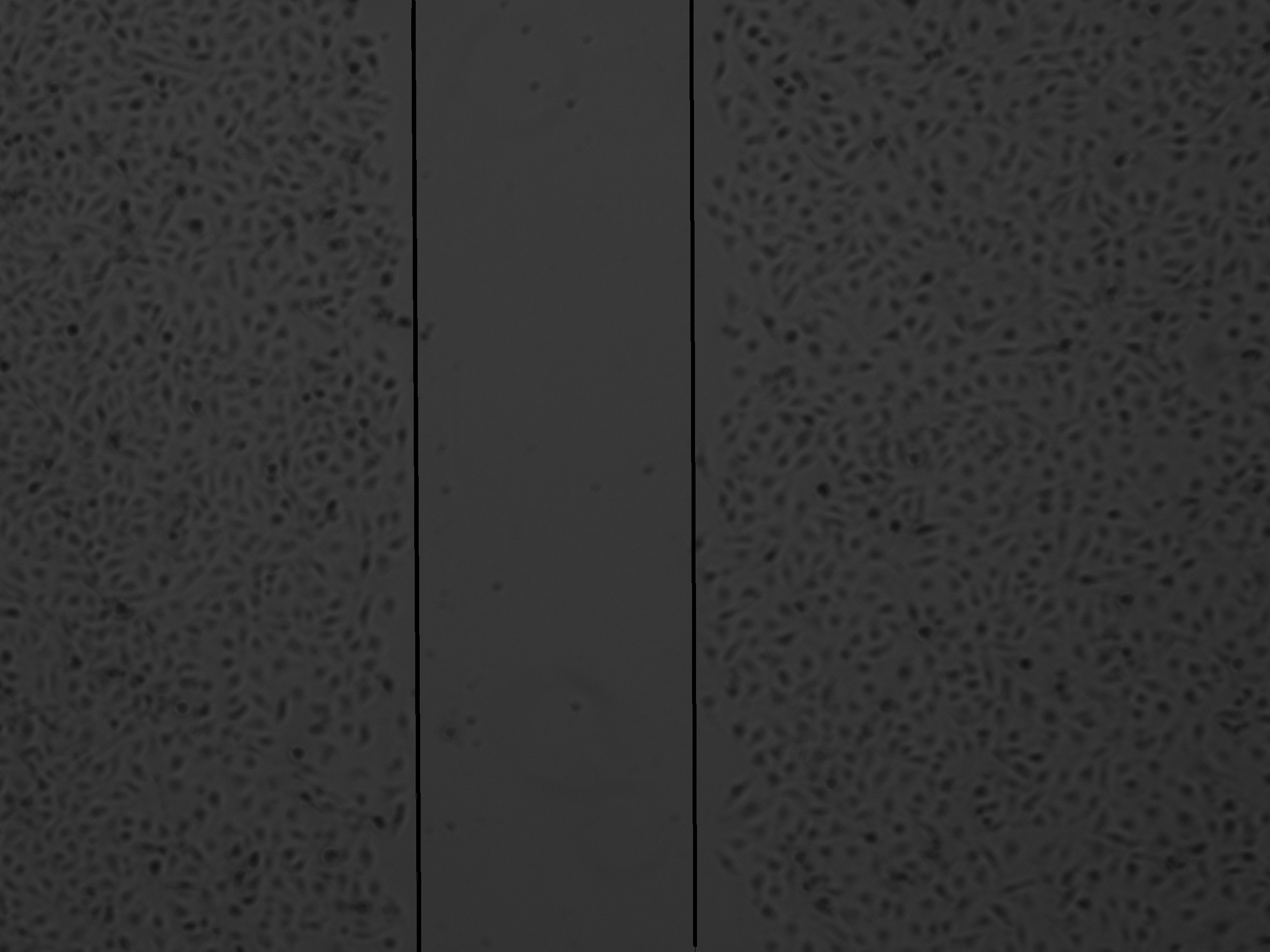

Supplement: Supplementary file 1 — Additional file 1. Basic clinical patient information. [file 12951_2023_2076_MOESM1_ESM.zip › oe circ_0001785+miR-513a-5p 24h.png]

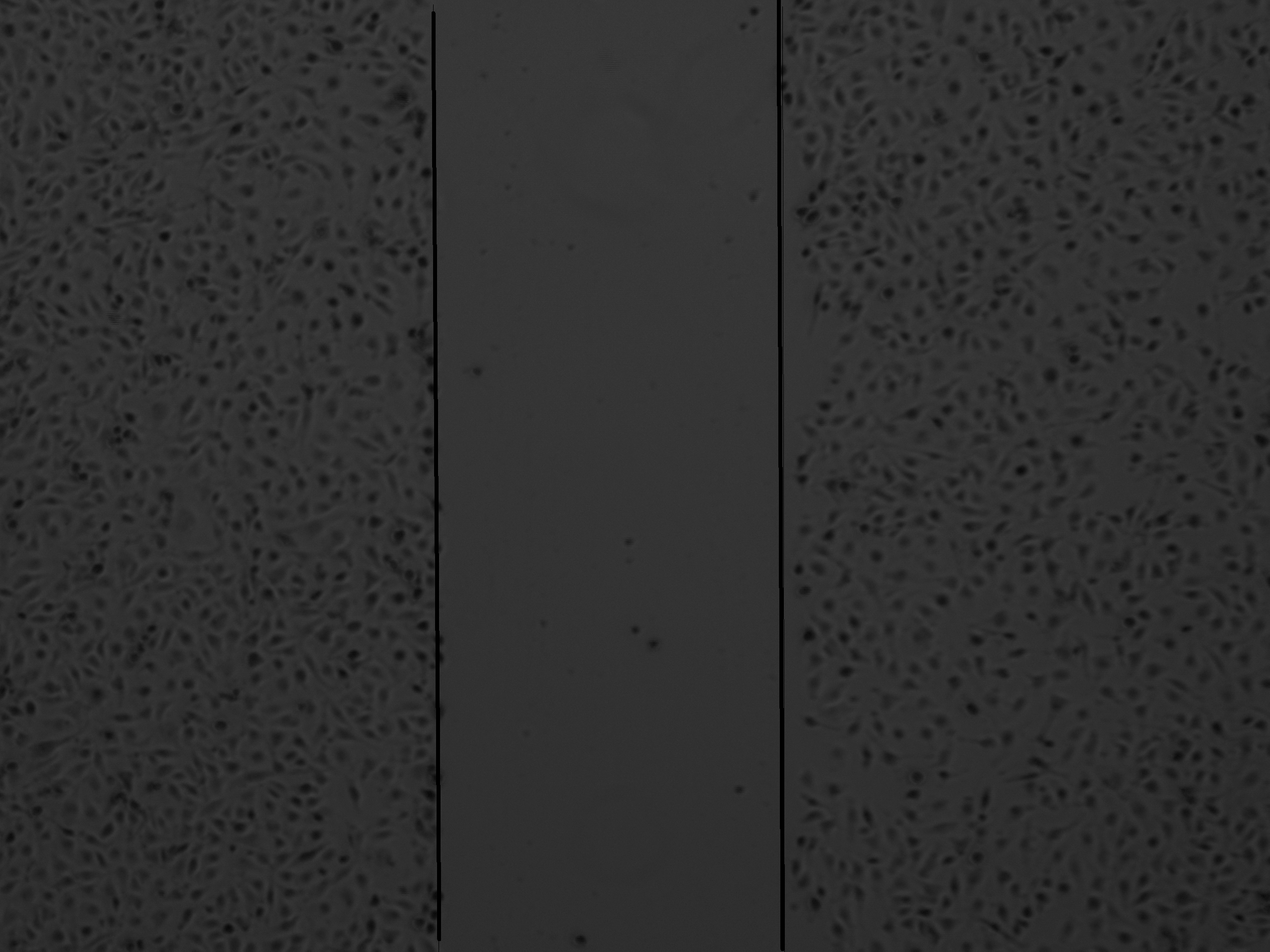

Supplement: Supplementary file 1 — Additional file 1. Basic clinical patient information. [file 12951_2023_2076_MOESM1_ESM.zip › oe circ_0001785+miR-513a-5p.png]

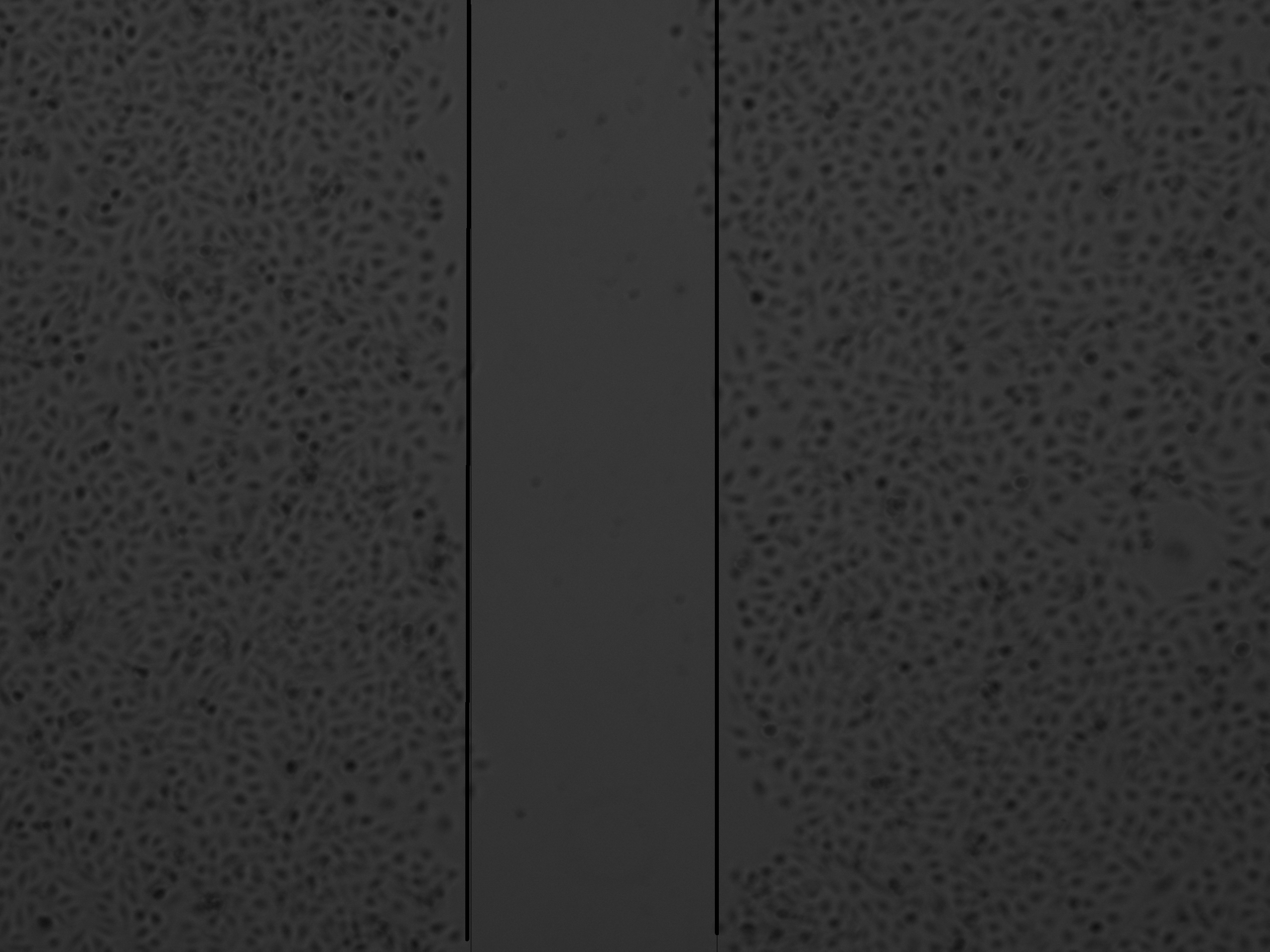

Supplement: Supplementary file 1 — Additional file 1. Basic clinical patient information. [file 12951_2023_2076_MOESM1_ESM.zip › oe circNC+miR NC 24h.png]

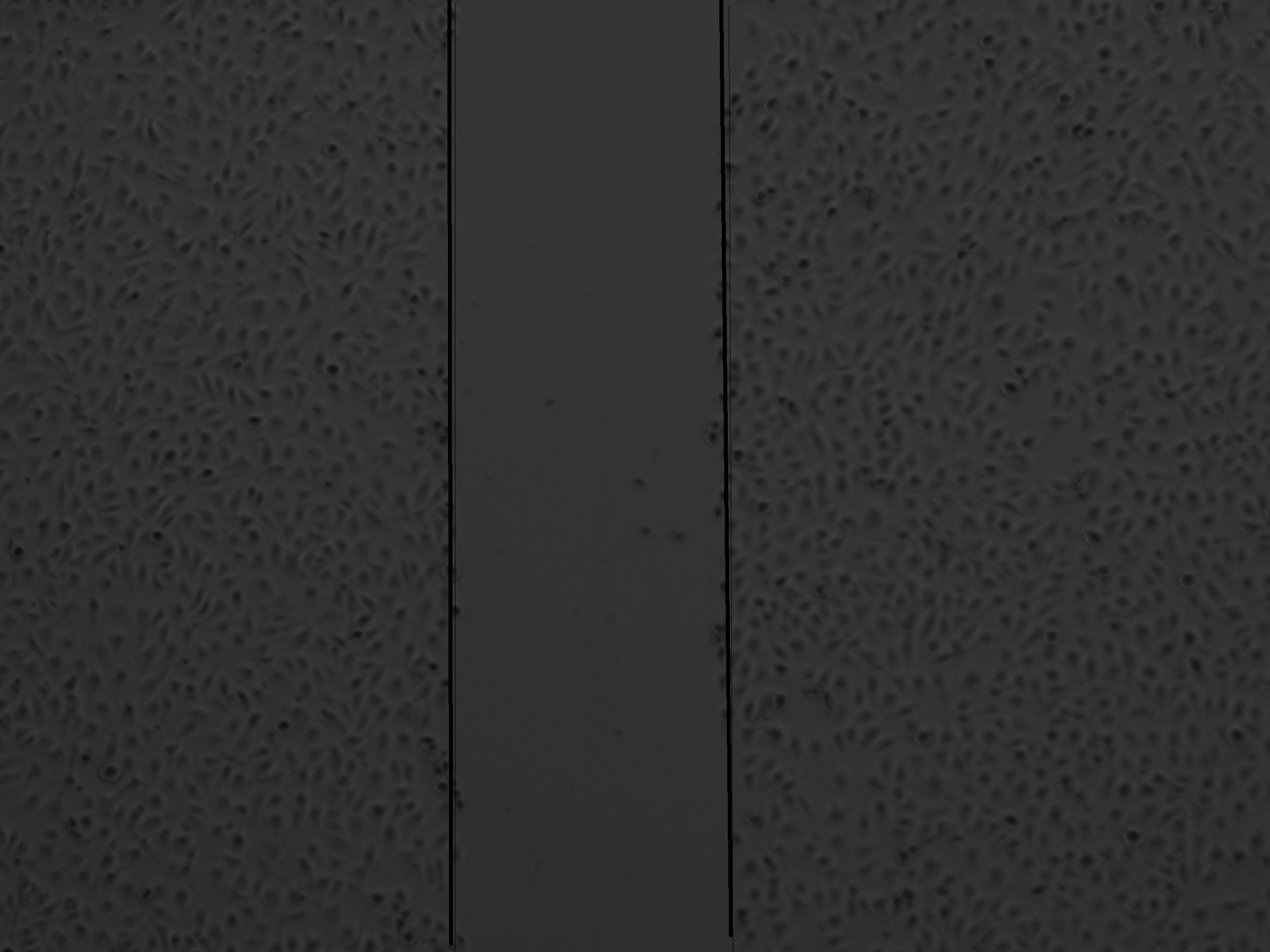

Supplement: Supplementary file 1 — Additional file 1. Basic clinical patient information. [file 12951_2023_2076_MOESM1_ESM.zip › oe circNC+miR NC.png]

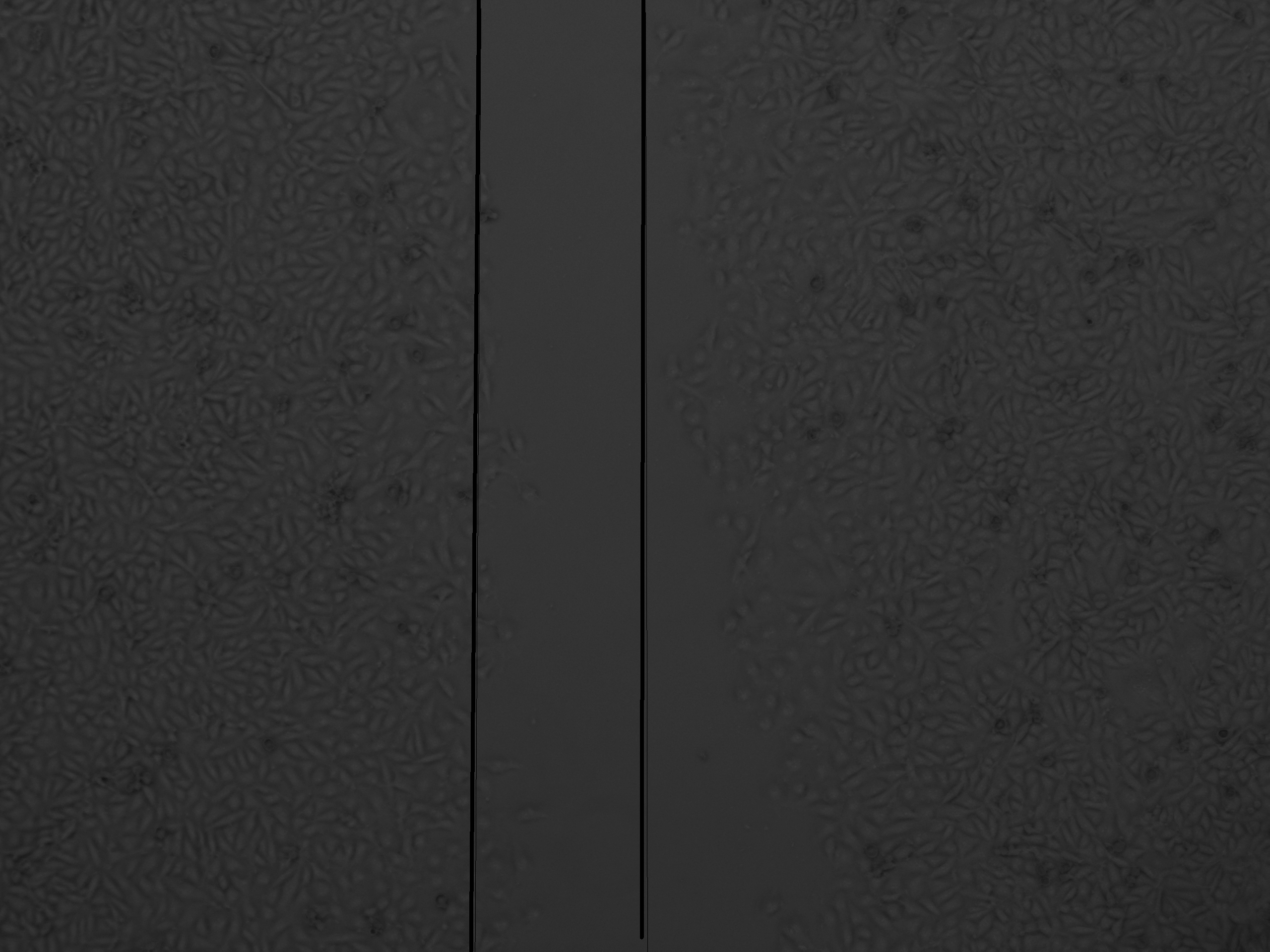

Supplement: Supplementary file 1 — Additional file 1. Basic clinical patient information. [file 12951_2023_2076_MOESM1_ESM.zip › oe circNC+miR-513a-5p 24h.png]

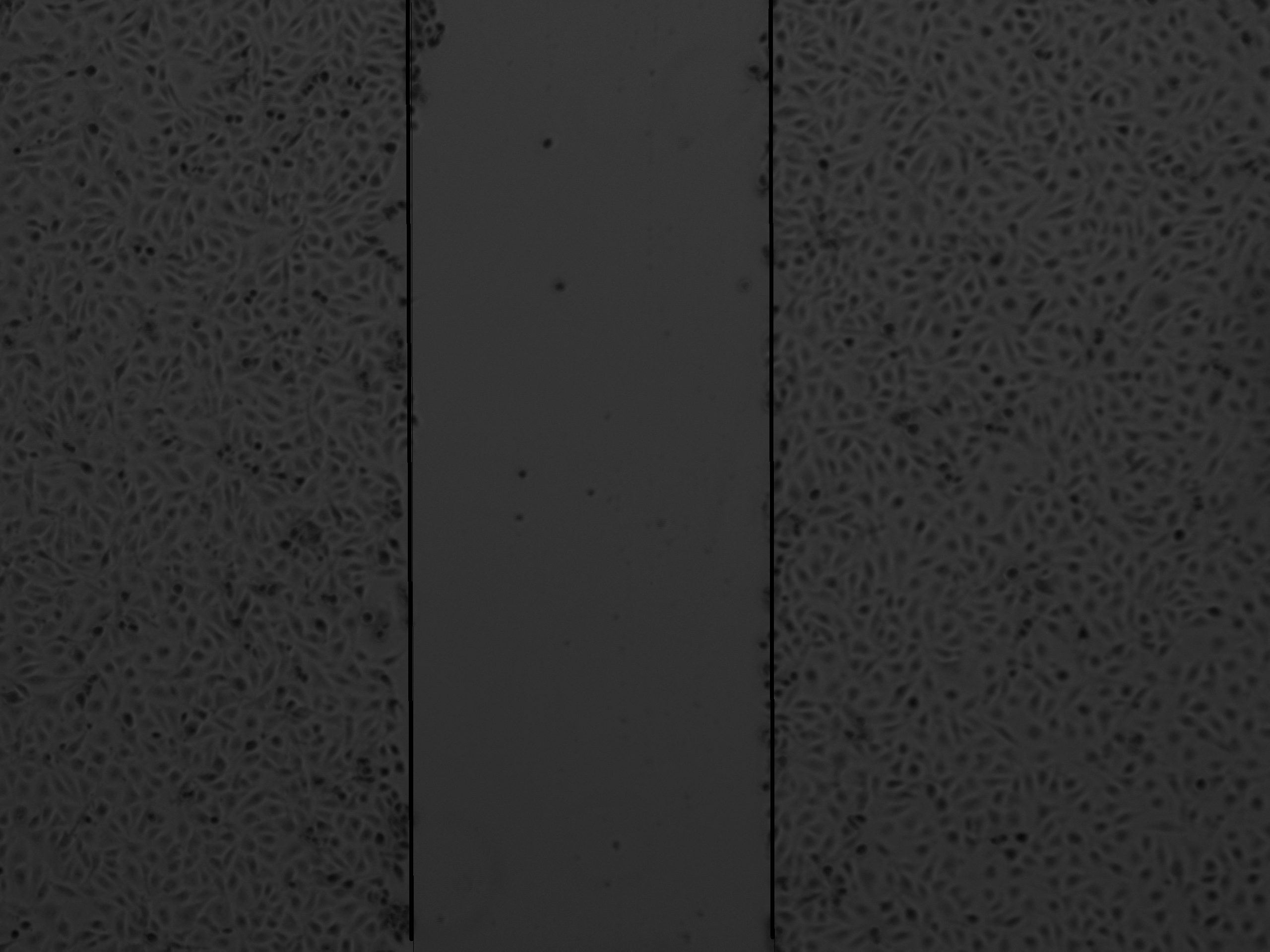

Supplement: Supplementary file 1 — Additional file 1. Basic clinical patient information. [file 12951_2023_2076_MOESM1_ESM.zip › oe_circ NC.png]

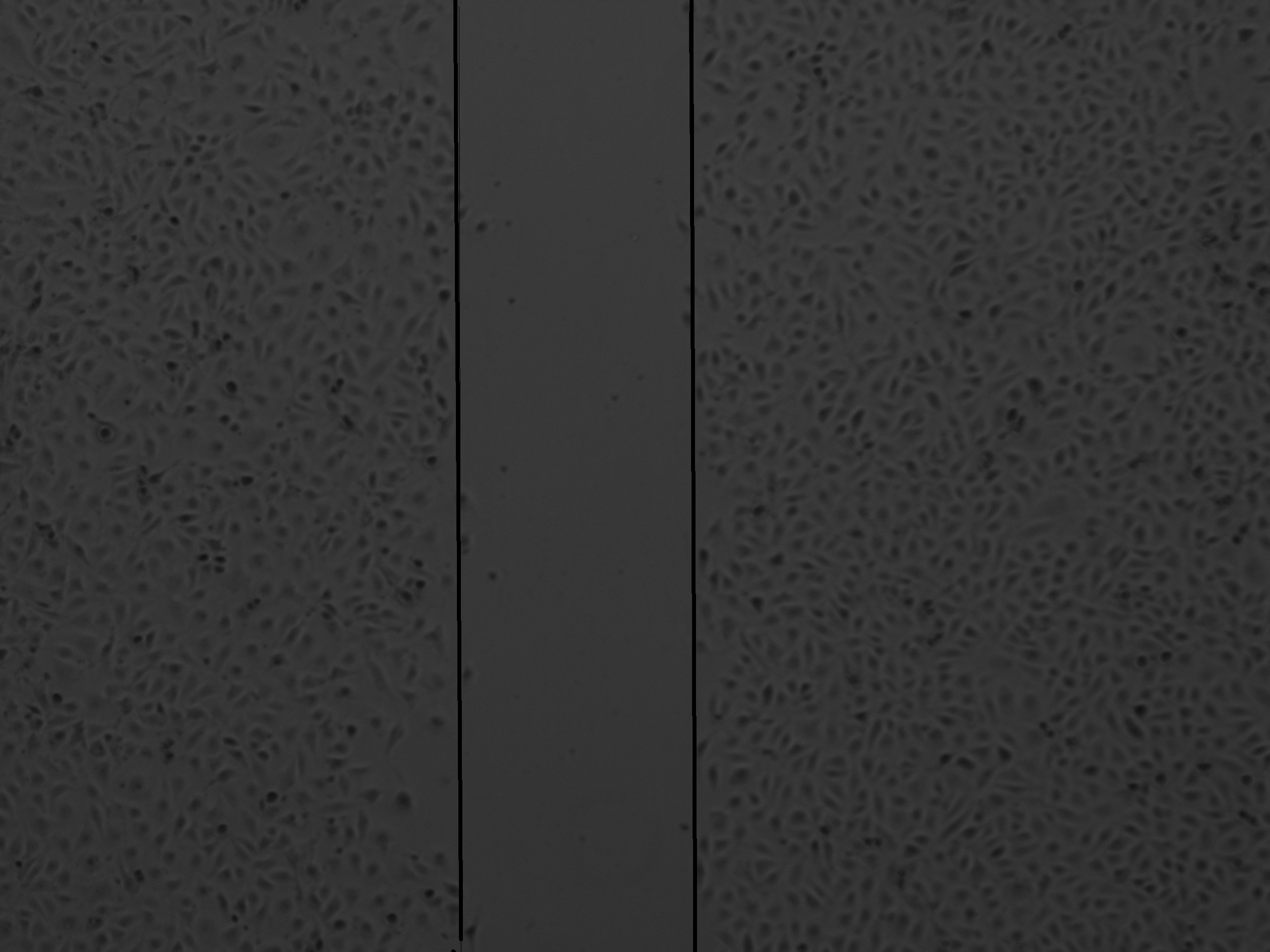

Supplement: Supplementary file 1 — Additional file 1. Basic clinical patient information. [file 12951_2023_2076_MOESM1_ESM.zip › oe_circ NC-24h.png]

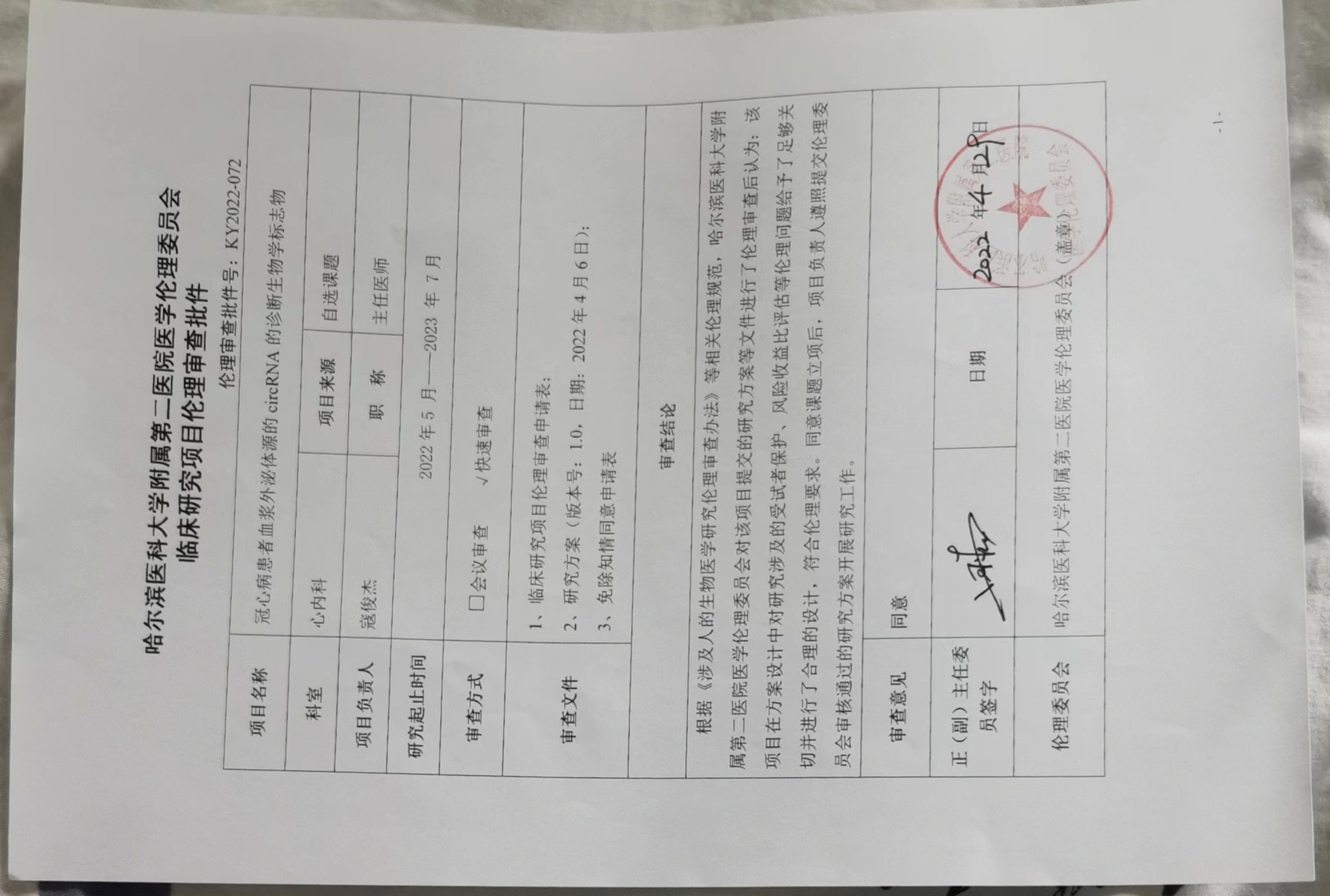

Supplement: Supplementary file 1 — Additional file 1. Basic clinical patient information. [file 12951_2023_2076_MOESM1_ESM.zip › Fig.jpg]

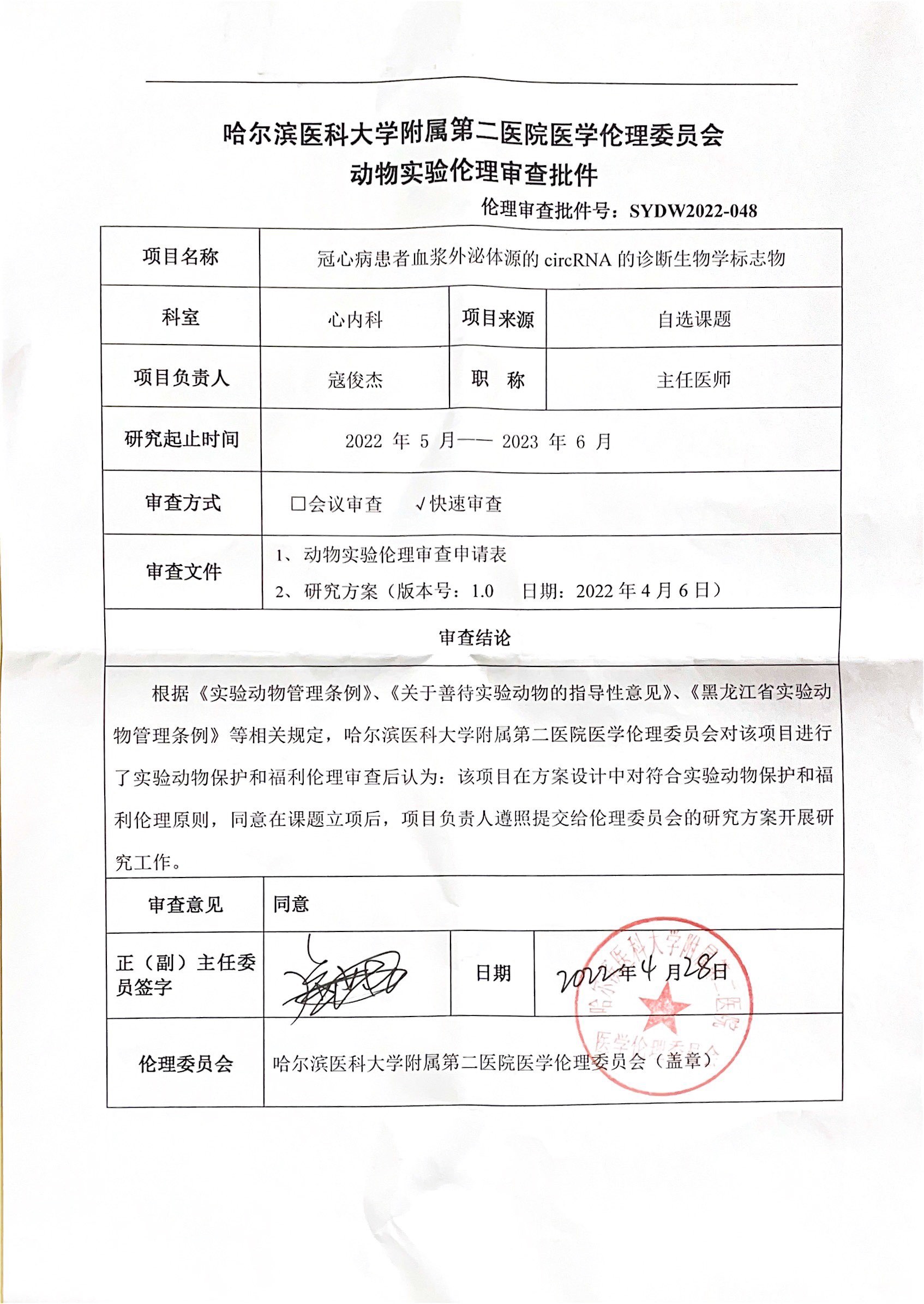

Supplement: Supplementary file 1 — Additional file 1. Basic clinical patient information. [file 12951_2023_2076_MOESM1_ESM.zip › Fig.tif]
