# Supplementary material for: Exosome-derived circ_0001785 delays atherogenesis through the ceRNA network mechanism of miR-513a-5p/TGFBR3
Source: J Nanobiotechnology. 2023 Oct 4;21:362. doi: 10.1186/s12951-023-02076-x (PMC10548746; doi:10.1186/s12951-023-02076-x)
Supplement: Supplementary file 2 — Additional file 2. The primer sequence of qRT-PCR test. Table S1. Basic clinical patient information. Table S2. The primer sequence of qRT-PCR test. [file 12951_2023_2076_MOESM2_ESM.docx]

Additional file

| Characteristic | CHD(*n*=31) | control(*n*=24) |
| --- | --- | --- |
| Age, y | 62.68±11.27 | 49.96±15.34 |
| Gender, M/F | 21/10 | 15/9 |
| Total cholesterol (mmol/L) | 4.208±0.9917 | 4.391±1.09 |
| Triglycerides (mmol/L) | 1.566±0.6568 | 1.42±0.6318 |
| LDL (mmol/L) | 2.555±0.1622 | 2.817±0.1952 |
| D-dimer (ng/ml) | 139.9±95.87 | 164.4±252.7 |
| Fibrinogen (g/L) | 3.003±0.7533 | 2.706±0.4012 |
| APTT (sec) | 40.36±28.17 | 34.45±2.637 |
| PT (sec) | 10.87±0.6503 | 12.59±3.381 |
| Platelets (×10^9^/L) | 220.9±54.27 | 209.6±55.64 |
| Leukocytes (×10^9^/L) | 8.152±3.101 | 6.988±2.094 |
| Neutrophils (%) | 71.44±2.211 | 70.99±1.832 |
| Monocytes (%) | 2.871±1.274 | 2.963±1.557 |
| Lymphocytes (%) | 23.94±1.866 | 24.92±1.57 |
| Previous PCI (n) | 23 | 0 |
| Hypertension (n) | 17 | 6 |
| Diabetes mellitus (n) | 10 | 2 |
| Cigarette smoking (n) | 7 | 5 |
| PRO-BNP (pg/ml) | 1215±2077 | 3337±9555 |
| Uric Acid (umol/L) | 342.1±98.42 | 325.2±105.5 |
| glycosylated hemoglobin (%) | 7.133±1.829 | 5.774±0.7723 |

**Table S1. Basic clinical patient information.**

Gender, age, diagnosis, hypertension, diabetes, uric acid, white blood cell, neutrophil, glucose and prothrombin time were ordered listed in this table.

| Gene name | Species | 5’-3’ |
| --- | --- | --- |
| circ_0001785 | F | ATTTCAGCATCAGGGATTTGGC |
|  | R | GCTCTCTCAGGTTACCATGCTC |
| β-actin | F | CTCCATCCTGGCCTCGCTGT |
| TGFBR3  U6  miR-513a-5p | R  F  R  F  R  RT  F  R  RT | GCTGTCACCTTCACCGTTCC  CCTAAGTGTGTGCCTCCTGA  CAATGCCCATCACGGTTAGG  CTCGCTTCGGCAGCACATATACT  ACGCTTCACGAATTTGCGTGTC  AAAATATGGAACGCTTCACGAATTTG  GCGCGTTCACAGGGAGG  AGTGCAGGGTCCGAGGTATT  GTCGTATCCAGTGCAGGGTCCGAGGTAT  TCGCACTGGATACGACATGACA |

**Table S2. The primer sequence of qRT-PCR test.**

Primers used in qRT-PCR tests are ordered and listed in this table.

**Methods**

**RNA extraction**

**Cellular RNA**

1. Take the cells from one well of the 6-well plate, discard the culture medium, and add 1 ml of trizol. Blow the cells off the wall of the culture dish with a pipette and transfer them to a new 1.5 ml EP tube to allow the cells to lyse fully.
2. Add chloroform in the ratio of 5 : 1 to 1.5ml EP tube without enzyme (e.g. trizol 1ml, chloroform 200ul). Mix thoroughly and let stand at 4°C for 5min, 4°C 12000rpm*15min.
3. After centrifugation, take an appropriate amount of colorless aqueous phase liquid (about 400 ul of supernatant) from the EP tube, add isopropanol at a ratio of 1: 1. Mix well, let it stand for 5min at 4°C, and 12000rpm*10min at 4°C. discard the supernatant, and the remaining precipitate is RNA.
4. Wash the precipitate with 1ml of 75% ethanol and leave it for 2min. 4℃ 7500rpm*5min, pour off the ethanol. Wash again with the same conditions of ethanol once.
5. After washing the ethanol for the last time, invert the EP tube in the air to dry the RNA. 5 min later dissolve the RNA with 20 ul of enzyme-free dd H2O (DEPC water) and assay the concentration. The amount of RNA was added uniformly to a concentration of 2000/RNA. The RNA was then reverse transcribed to cDNA using the RNA Reverse Transcription Kit according to the instructions. or directly store the RNA at -80°C.

**Tissue RNA:**

Human plaque tissues and mouse tissues were stored in a -80°C refrigerator prior to tissue removal. Approximately 0.1 g of each tissue sample was removed and immediately placed in an ice box. Immediately afterward, 2 ml of EP tubes bearing tissue and magnetic beads were placed into a tissue grinder with the condition set to 600 rpm*60s to grind the tissue to a powder. The tissue was then transferred into a 1.5 ml centrifuge tube to which 1 ml of trizol had been added, shaken, rested, and centrifuged. After centrifugation, the supernatant was transferred into a new centrifuge tube. The rest of the test was performed for cellular RNA.

**Human blood RNA**

Collect 3 ml of whole blood using EDTA anticoagulation tube, then add erythrocyte lysate in the ratio of 1:3 (Example: 3 ml of whole blood, 9 ml of erythrocyte lysate) into a 15 ml EP tube. Mix gently upside down for several times and let stand at room temperature for 5-10 min at 2500 rpm*5 min. Discard the supernatant, aspirate the supernatant as much as possible, then add 1 ml of PBS and wash by centrifugation at 2500 rpm*5 min. The supernatant was discarded, which means 80% of leukocytes were obtained. Subsequently, 1ml of trizol was added and the cellular RNA extraction procedure was repeated.

**Reverse Record**

The Roche Reverse Transcription Kit was used. We used a 20ul reverse transcription system and added the required enzymes, primers, and total RNA to the tubes according to the instructions, and finally added enzyme-free ddH2O to replenish the solution to 20μl. Place the samples to be reverse transcribed in a centrifuge at 300 rpm*10 s and put them on the machine. If not used, store the reverse transcribed RNA in a refrigerator at -20°C.

The reverse transcription system is as follows.

①0.5u ②4u ③0.5u ④2u ⑤1u , total 8u

For example, the RNA concentration is 300, that is, the amount of RNA added: 2000/300 (concentration) ≈ 6.7u

⑦DEPC: 20-8-6.7 = 5.3u , total 20u system

On-boarding procedure.

First, add RNA and ⑤ (reverse transcription of miR is added with miR exclusive reverse transcriptase) and DEPC water to 13ul, and then on the machine. The program is 65℃*10min, 4℃*5min.

Then add ①②③④ to the EP tube respectively and get on the machine. The procedure is

①50℃*30min ②25℃*10min ③85℃*5min ④4℃*5min ⑤ END

**Real-time fluorescence quantitative PCR**

The upstream and downstream primers for the desired target molecules (circ0001785,miR-513a-5p, TGFBR3), SYBR Green, cDNA, and the system supplemented with DEPC water to 10 μl were added according to the conditions required for SYBR Green. Centrifuge at 1000rpm*60s in a centrifuge. Set the temperature and cycle number according to the SYBR Green instruction manual and get on the machine. After finishing, the relative levels of the desired gene expression were calculated based on the resulting CT values.

Configuration method.

1. SYBR 5μl ②Pre-primer 0.4μl ③Post-primer 0.4μl ④DEPC water 2.2μl ⑤cDNA 2μl

The number of temperature cycles.

①Pre-denaturation 95℃*30s ②95℃*10s 45 cycles of reaction ③60℃*30s ④End

The expression levels of circRNA and mRNA were analyzed using β-actin as an internal reference. miRNA expression levels were analyzed using U6 as an internal reference. The relative expression levels of these genes were calculated by the 2^-△△CT^ method. Normal distribution analysis of the data was performed by GraphPad prism software. The t-test was used for data conforming to a normal distribution, and the Wilcoxon paired signed-rank test was used for data not conforming to a normal distribution. Thus, the relative expression levels of genes in blood and plaque tissues of patients with coronary artery disease and normal patients were compared.

**Cell Plasmid Transfection**

To overexpress miR-513a-5p, we synthesized a miRNA mimic. miRNA mimic and the negative control sequence of mimic (mimic-NC) were transfected into cells according to the instructions for the Lipo8000™ transfection reagent. transfection was terminated after 48 hr.

The steps are as follows.

① Discard the old medium and rinse the cells 3 times with PBS. Then add fresh complete medium.

1. Configure the amount for one well of the six-well plate. Configure the mixture of transfection reagent and miRNA. Take a sterile centrifuge tube, add 125 ul of high sugar DMEM cell medium, and 100 pmol of miR-513a-5p mimic or mimic NC. mix gently with a pipette, then add Lipo8000™ reagent and mix gently again. Incubate at room temperature for 20 minutes.
2. Gently and uniformly drop the incubated miR-513a-5p mimic or mimic NC and Lipo8000™ mixture into the cell culture in step "②". Incubate for 48 hours to complete transfection.

**Cellular lentivirus transfection**

To overexpress circ0001785, we synthesized the overexpressed hsa_circ_0001785 at Hanheng with the following sequence.

>hsa_circ_0001785|NM_018091|ELP3，length=467bp，%GC=45.82

GGATATTCCAATGCCTTTAGTTAGCTCAGGAGTAGAGCATGGTAACCTGAGAGAGCTGGCACTTGCAAGAATGAAAGACC

TCGGAATACAGTGTCGAGATGTGAGAACCAGAGAAGTTGGAATCCAAGAAATTCATCACAAAGTACGGCCATACCAGGTT

GAATTGGTAAGGAGAGATTATGTTGCAAATGGTGGCTGGGAAACATTCTTGTCATACGAAGACCCAGATCAAGACATTTT

GATTGGCCTCCTACGATTACGCAAGTGTTCAGAAGAAACTTTCCGTTTCGAATTGGGTGGAGGTGTCTCCATAGTACGAG

AGCTGCATGTGTATGGGAGTGTGGTCCCTGTGAGCAGCCGGGATCCTACTAAATTTCAGCATCAGGGATTTGGCATGCTG

CTGATGGAGGAAGCAGAAAGAATAGCTAGAGAAGAACATGGGTCTGGGAAAATCGCTGTGATATCAG

1. Optimal MOI groping.

1) Cells to be transfected are counted and spread into 24-well plates, and transfection of the virus is started when cell density is 70%.

2) Set different MOI for infection.

3) Observe the cell status and choose the best MOI for the formal experiment, the best MOI in this experiment is 30.

1. Dilute the lentivirus with a complete medium.
2. Add the virus to the cells according to the optimal MOI.
3. Observe the cell status after 6 h and replace the complete medium again.
4. Perform subsequent experiments after 72h-96h of infection.

**Dual luciferase reporter experiment**

Experimental groups.

1. Blank experimental group.
2. Target gene circRNA0001785 wild-type dual luciferase reporter vector + miR-513a-5p mimic group.
3. Target gene circRNA0001785 wild-type dual luciferase reporter vector + mimic NC group.
4. Target gene circRNA0001785 mutant dual luciferase reporter vector + miR-513a-5p mimic group.
5. Target gene circRNA0001785 mutant dual luciferase reporter vector + mimic NC group.
6. Target gene TGFBR3 wild-type dual luciferase reporter vector + miR-513a-5p mimic group.
7. Target gene TGFBR3 wild-type dual luciferase reporter vector + mimic NC group.
8. Target gene TGFBR3 mutant dual luciferase reporter vector + miR-513a-5p mimic group.
9. Target gene TGFBR3 mutant dual luciferase reporter vector + mimic NC group.

These cDNA fragments were inserted into the vector after the synthesis of the above genes. HUVEC cells (1×10^4^ cells/well) were inoculated into 96-well plates. and cotransfected with Lipo8000™. After transfection for 48h, the fluorescence intensity was detected by luminescence zymography according to the instructions for use of the dual fluorophore enzyme reporter gene assay reagent. The brief operation steps are as follows.

1. Complete transfection of the cells in a 96-well plate.
2. Remove the 96-well plate and relieve it at room temperature for 15 min.
3. Mix well and let it stand at room temperature for 10 min. then detect the chemiluminescence using luminescent zymography.
4. Take 100μl of sea kidney fluorophore luciferase reagent dissolved in advance at room temperature (1:100 ratio configuration).
5. Mix well and allow to stand for 10 min. subsequently, detect the chemiluminescence using luminescence zymography.

**Wound healing assay**

Cells that have been transfected with lentivirus or plasmid are spread evenly in 6-well plates, respectively. 24 hours later the cells are cultured to approximately 80-90% density. Gently trace a straight line on the cell monolayer with the tip of a sterile 200 μL pipette. This creates a uniform width "scratch". Then place an appropriate amount of serum-free DMEM medium in each well and continue to incubate. The cultured cells were photographed at 0 hr, 6 hr, and 24 hr, respectively.

**Cell proliferation assay**

**CCK-8 assay.**

For CCK-8 experiments, first, we inoculated cells (1.0×103 cells/well) in 96-well plates. After the cells were allowed to adhere to the wall, they were treated with drugs and fluid changes. After incubating again for 12-24 hours, 10uL of CCK-8 reagent was mixed with 90uL of the complete medium into each well at a predetermined time point. Incubate for 30 minutes in a cell incubator under a light-proof environment. Finally, the OD value of each well was measured at 450 nm wavelength on an enzyme marker.

**EdU experiment.**

Cells (7.0*103 cells/well) were uniformly inoculated in 96 plates. Allow 24 hours for the cells to adhere and stabilize before adding EdU working solution to each well and incubating for 2 hours. Then follow the procedure of BeyoClick™EdU-484. Finally, the samples are photographed by inverted fluorescence microscopy. The detailed steps are as follows.

1. Incubate 2X of EdU reagent at 37°C. Then add the cell culture medium to the EdU reagent in a 1:1 ratio to make the final concentration of EdU in the 96-well plate half of the original.
2. Continue to incubate the cells in the cell culture incubator for 2 hours.
3. The cells were fixed with 4% paraformaldehyde and left at room temperature for 15 min.
4. Pour off paraformaldehyde and rinse 3 times with PBS for 5 min each time.
5. Carefully aspirate PBS, add 0.1% TritonX-100 to break the membrane permeabilization and leave for 15 min at room temperature.
6. Discard the permeabilization solution. Wash the cells with PBS 3 times for 5 min each time.
7. Add the appropriate amount of Click reagent according to the instructions of Beyoncé and leave for 30 min at room temperature under shade.
8. Wash with PBS three times, then add Hoechst reagent and leave for 15 min at room temperature under shade.
9. Wash with PBS for 5 min each time and wash 3 times, observe with an inverted fluorescence microscope, and take photos.

**PI + Hoechst assay**

For the PI+hoechst apoptosis assay, we first inoculated cells transfected with plasmid or lentivirus (1.0×10^3^ cells/well) in 96-well plates overnight. The cells were gently washed 3 times with PBS for 5 min each time after the cells were attached to the wall, and then 100 ul of mixed PI+hoechst mixture (PI=1:100, hoechst=1:1000) was put into each well. The wells were placed in a refrigerator at 4°C for 30 minutes, followed by 2 washes with PBS for 5 minutes each, and finally photographed using an inverted fluorescence microscope.

**Animal HE staining.**

1. After taking paraffin sections of mouse heart aortic sinus tissues, dewaxing and overwaxing were performed first, and the steps were the same as above.
2. Wash with PBS 3 times for 5 min each time.
3. Hematoxylin stain was drip-stained for 5 min, followed by rinsing with running water for 15 min.
4. Next, use eosin stain to drip stain for 3min and wash again with running water to remove the excess stain.
5. Finally, alcohol was used to dip wash from low to high gradient (75% alcohol, 95% alcohol, 100% alcohol for 10s each).
6. Xylene was dehydrated twice for 1 min each, and the slices were sealed. Observe the tissue under the microscope.

**Animal Masson staining.**

1. After taking paraffin sections of mouse heart aortic sinus tissue, dewaxing, and overwaxing were performed first, and the procedure was the same as above. Finally, the sections were washed 3 times with PBS.
2. Put the sections into the mordant staining solution for mordant staining, incubate in a 57°C oven for 1h, and then rinse with water for 15min.
3. Lichon red magenta staining solution was drip-stained for 15 min, and distilled water was washed twice for 30 s each time.
4. Followed by alcohol gradient dehydration, 100% alcohol, 95% alcohol, and 85% alcohol for 5s each.
5. Use phosphomolybdic acid staining solution to stain dropwise for 5min. discard the upper solution, the sections are not washed, and aniline blue staining solution is added dropwise directly for 4min. put into ddH_2_O for 5s to wash away the excess stain. Xylene was used twice for 30 s. Neutral gum was used to seal the slices.

**Animal immunohistochemistry.**

1. After taking paraffin sections of mouse heart aortic sinus tissue, dewaxing, and overwaxing were performed first, and the procedure was the same as above. Finally, the sections were washed 3 times with PBS.
2. Take out the sections, firstly in an autoclave, add EDTA antigen repair solution PH6.0 (citrate repair solution), place the sections in it, and allow the repair solution to completely soak the tissue. Cover the pot, wait for the pressure valve of the autoclave to rotate completely to the lowest point, and deflate and remove the sections after pressure reduction. Set aside to cool at room temperature.
3. After cooling to room temperature, wash 2-3 times for 5 min each using PBS buffer soak.
4. Next, add the appropriate amount of endogenous peroxidase blocker and incubate at room temperature for 10 min. rinse with PBS buffer for 3 min, 3 times consecutively.
5. Incubate with 0.1% TritonX-100 for 15 min at room temperature and wash with PBS for 5 min each time 3 times.
6. Subsequently closed with 5% BSA bovine serum for 30min in a dressing box at 37°C.
7. Discard BSA after 30min, add primary antibody: CD31 (1:200), and place in a dressing box at 37°C for 1 hour.
8. Subsequently wash with PBS for 5min each time, 3 times.
9. Add matching immunohistochemical secondary antibody (1:200) and incubate at 37°C for 30 min.
10. Discard the secondary antibody and wash again with PBS for 5 min each time, 3 times.
11. Configure the DAB color developer, add the appropriate amount of freshly prepared DAB color developer, and incubate for 5 min at room temperature.
12. Next, rinse with tap water, incubate with hematoxylin staining solution for 30 seconds, and rinse back to blue for 10 min.
13. Lastly, wash with alcohol in a gradient from low to high (75% alcohol, 95% alcohol, 100% alcohol for 10s each), dehydrate twice with xylene for 1min each, and seal the film.
14. Observe the tissue under microscope.
